# New Trends on Sensing - Monitoring - Telediagnosis for Life Sciences Bucharest, România: NT-SMT-LS September 7-9, 2017

**Published:** 2017

Leland C. Clark can be considered as the father of biosensor concept. He is the pioneer in this field to design an intelligent electrochemical sensors based on enzyme as bio-recognition element. Since then, many researchers worked on the development of biosensors for wide spread applications including but not limited to medical, agro-food and environmental interests. The research output was very promising, and as a result, governmental and private companies invested and promoted research in the biosensor field. The number of publications increased exponentially and “start-up” companies were created to transfer the technology from laboratory to commercial applications.

Recent years have witnessed an increasing demand of biosensors for medical applications. Commercial biosensors have been developed for the detection of glucose for diabetes people, lactate sensor for sportsmen and urea sensor to monitor dialysis. It is anticipated that in the coming years, scientists will focus to monitor the human body in all aspects: livers will be monitored to make sure that enzymes are functioning correctly in filtering out the toxins, hearts will be monitored to avoid the heart attacks, cancer will be detected in its earliest stages and individual cancer cells may even be killed using another portion of the sensor. Indeed the body will be monitored continuously to determine possible health concerns that may arise

