# Supplementary material for: New Trends on Sensing - Monitoring - Telediagnosis for Life Sciences Bucharest, România: NT-SMT-LS September 7-9, 2017
Source: J Med Life. 2017;10(Spec Iss 2):1–33. (PMC6088551)
Supplement: Related texts

# Meeting report: K.N.1.2. THIN FILMS FABRICATED BY PULSED LASER TECHNOLOGIES FOR BIOSENSING

**Authors:** I.N. Mihailescu, L. Floroian, C. Ristoscu

**Affiliations:** 1 National Institute for Lasers, Plasma and Radiation Physics, Magurele, Romania;; 2 Faculty of Electrical Engineering and Computer Science, Transilvania University of Brasov, Romania

BIOSENSORS are powerful tools designed to provide selective identification of toxic chemical compounds at ultra-trace levels in industrial products, chemical substances, environmental samples or biological systems for biomedical diagnosis.

We review new results in the pulsed laser synthesis of nanostructured thin films for use in biosensing.

Pulsed laser deposition (PLD), Matrix-assisted pulsed laser evaporation (MAPLE) and Laser direct write (LDW) proved some competitive method to grow high quality thin films.

PLD growth of undoped and Au-doped ZnO coatings on SiO2 substrates along with detection test results for different concentrations of butane in dry air and nitrogen by the m-lines technique is reviewed. Effective index variations down to 0.005 were detected during butane exposure at room temperature. The obtained structures preserve gas sensitivity down to 20 °C. Improvements are expected by elimination of the losses in light coupling mismatch of investigated films and miniaturization.

We immobilized by MAPLE urease in form of thin films and showed that it is active in breaking down and diagnose of urea content in blood. MAPLE deposited IgG coatings could be used as immunosensors for the recognition of specific antigens in research, as well as in clinical investigations. It stands, in our opinion, for an essential step for the development of personalized, miniaturized biosensors.

We concluded that the thin films deposited by pulsed laser techniques were identical in composition, morphology and structure with the base material, and most likely preserved their functionality.

# Meeting report: KN.1.3. DESIGNING AND CONDUCTING EPIDEMIOLOGICAL RESEARCH; CARDIOMETABOLIC EXAMPLES FROM A LARGE U.S. COHORT: THE JACKSON HEART STUDY

**Authors:** A. Bidulescu

**Affiliations:** * Indiana University Bloomington, Department of Epidemiology and Biostatistics, Bloomington, IN

Adipose tissue has emerged as a metabolically active participant in mediating vascular and myocardial dysfunction, primarily by release of several paracrine and endocrine bioactive peptides termed adipokines that modulate appetite (leptin, ghrelin), glucose metabolism (adiponectin, resistin, leptin), bone metabolism (leptin, adiponectin), vascular tone (angiotensin, ghrelin, brain natriuretic peptides), and vascular structure (angiotensin, leptin, adiponectin). Several adipokines have been implicated in the pathobiology of atherosclerosis and heart remodeling, and changes in their synthesis and secretion may contribute to development of obesity and ultimately to atherosclerosis, stroke and heart failure. Importantly, although the protective adipokine adiponectin is inversely associated with atherosclerosis, its relationship with CVD events varies between populations and is dependent on cardio-metabolic conditions. Our studies suggest that AA have lower levels of adiponectin compared to EA. In our Jackson Heart Study ancillary investigations, we demonstrated 1) an inverse relationship between serum adiponectin and left ventricular (LV) mass only in normotensive and non-insulin resistant participants, suggesting that adiponectin may interact with LV mass in mediating long term CVD risk; 2) that adiponectin was inversely associated with visceral adipose tissue (mediated through insulin resistance) and directly associated with abdominal subcutaneous adipose tissue thus confirming the putative favorable/buffering effect of abdominal subcutaneous tissue; 3) that AA without obesity or insulin resistance exhibit a positive association between global individual EA ancestry and adiponectin levels, that is abolished in the setting of increased adiposity; and 4) that adiponectin levels were directly associated (instead of inversely) with incident stroke among women, even after accounting for insulin resistance and hypertension status. This suggests that visceral adiposity is a key regulatory mechanism governing the body’s metabolism and an adaptive/feedback source of key adipokines such as adiponectin. It also raises the intriguing research question of whether adiponectin harbor harmful properties by itself, or it is produced in response to vascular inflammation to counter the atherosclerotic process, or the putative 'adiponectin resistance' phenomenon acts.

This presentation will highlight some methodological and scientific aspects of merging molecular and behavioral epidemiology tools in an effort to shed some light on a series of health disparities issues. A few cardio-metabolic examples will be used, examples very pertinent to the current obesity epidemics.

# Meeting report: KN.1.4. BETA-GLUCANS IN PREVENTION AND CONTROL OF METABOLIC SYNDROME

**Authors:** M. Shamtsyan

**Affiliations:** * St. Petersburg State Institute of Technology (Technical University), St. Petersburg, Russia

Metabolic syndrome is the name for a group of factors that raises risk for serious health problems, such as, diabetes, cardiovascular diseases, stroke and cancer. Metabolic syndrome is a combination of at least three of the five following medical conditions: abdominal obesity, elevated blood pressure, elevated fasting plasma glucose, high level of serum triglycerides, and low levels of high density lipoproteins.

Our studies demonstrate, that consumption of beta-glucans can significantly decrease the levels of serum triglycerides and increase the ratio of high density lipoproteins to low density lipoproteins. Beta-glucans were strongly decreasing elevated fasting plasma glucose levels in streptozotocin- induced diabetic rats. Also a significant immune-modulating action of beta-glucans was demonstrated. 

Recent studies show, that abdominal obesity induces a state of chronic, low-grade inflammation that facilitates to insulin resistance. Adipose tissue macrophages infiltrate adipose tissue during obesity and contribute to insulin resistance and pancreatic beta-cell disfunction. Beta-glucans, can decrease the insulin resistance through the regulation of various inflammatory cytokines and also can protect pancreatic- beta-cells.

# Meeting report: KN.1.5. STROKE THERAPY MONITORING USING TELEMETRY

**Authors:** A. Popa-Wagner

**Affiliations:** 1 Essen University Medical School, Germany; 2 University of Medicine and Pharmacy Craiova, Romania

In aged humans, stroke is a major cause of disability for which no neuroprotective measures are available. In adult rodents the endogenous neurogenesis is maintained in the subventricular zone and the dentate gyrus of the hippocampus and could be used to improve post-stroke outcome. However, recent work done in our research department revealed that most of the signalling pathways implicated in neurogenesis are downregulated in both age groups. 

We aimed at achieving neuroprotection by stimulating endogenous neurogenesis and diminishing inflammation and edema in aged animals after stroke

Aged rats were subjeted to stroke by cranioectomy and MCA occlusion. Lower body temperature was achieved with H2S. Main physiological parameters were measured telemetrically.

Behaviorally, hypothermia also had a limited beneficial effect. Finally, after H2S-induced hypothermia, the post-stroke aged rats experienced a persistent sleep impairment during their active nocturnal period.

Our data suggest that cellular events that are delayed by hypothermia in aged rats may, in the long-term, rebound and diminish the beneficial effects. However, as we have shown in this work, the beneficial effects of H2S may extend beyond the cooling effect.

A better understanding of the pathophysiology of the ischemic injury processes on which hypothermia and H2S act will serve to further promote the use of this promising method to reduce the mortality and morbidity caused by stroke.

# Meeting report: KN.1.6. ASSESMENT OF OXIDATIVE STRESS PARAMETERS IN DIFFERENT PATHOLOGIES

**Authors:** I. Stoian

**Affiliations:** * “Carol Davila” University of Medicine and Pharmacy, Bucharest, Romania

Oxidative stress is a common feature of a very large panel of pathologies. Several methods to assess the oxidant/antioxidant perturbances were developed but the exact clinical significance of these imbalances is missing. This can explain the poor performance or sometimes even the toxicity of antioxidants administration.

# Meeting report: KN.1.7. POLYPHENOL-RICH FOOD IN NEURODEGENERATION: MECHANISMS AND PREVENTION

**Authors:** R. F. M. Silva, L. Pogačnik

**Affiliations:** 1 Research Institute for Medicines (iMed.ULisboa), and Department of Biochemistry and Human Biology (DBBH), Faculty of Pharmacy da Universidade de Lisboa, Lisboa, Portugal.; 2 Biotechnical Faculty, Department of Food Science and Technology, Ljubljana, Slovenia

It is well known that in most neurodegenerative diseases, neurodegeneration occurs long before the onset of first symptoms. Any substance that can prevent such early neuron loss may delay, or prevent, neurodegenerative diseases. Many food products (e.g. fruits, vegetables, green tea, wine) are rich in polyphenols, that revealed to be potent antioxidants and share anti-inflammatory activity. Several studies using such polyphenols have been focused on their neuroprotective role in neurodegenerative diseases. It is now known that polyphenols modulate several cellular functions and molecular pathways. It was recently shown that curcumin reduces astrocyte production of TNF-α and IL-6 in an animal model of Parkinson’s disease and modulates both MyD88-dependent and TRIF-dependent pathways in TLR4 signaling. Also, EGCG has an anti-amyloid capacity acting as a ‘b-sheet breaker’ and it was shown that significant levels of EGCG can cross a human BBB model and protect neurons from oxidative cell death. EGCG can also inhibit Bax, cytochrome c translocation, and autophagic pathways, through increasing LC3-II and modulation of mitochondrial functions. As so, the enrichment of daily diet with food-rich polyphenols, or their controlled use as food supplements, increasing brain defenses, seems to represent a promising, widespread and relatively cheap approach to counteract neurodegenerative diseases.

*Work supported by Fundação para a Ciência e a Tecnologia (FCT), Portugal (UID/DTP/04138/2013) and Slovenian Research Agency (research core funding No. P4-0121)*.

# Meeting report: O.1.1. FAST SCREENING METHODS TO DETECT ANTIBIOTIC RESIDUES

**Authors:** G. Catanante, A. Sharma, J-L Marty

**Affiliations:** 1 BAE Laboratory, Université de Perpignan, Perpignan, France; 2 Biosensor Lab, Department of Chemistry, BITS, Pilani- K. K. Birla Goa Campus, Zuarinagar, Goa

Antibiotics are drugs of natural, semi-synthetic or synthetic origin. All antibiotic families used today for the treatment and prevention of bacterial diseases were first discovered prior to the mid 1980s. Antibiotics revolutionized medicine in the 20th century, and have led to the near eradication of diseases such as tuberculosis in the developed world. However, the overuse and misuse of antibiotics especially in husbandry, as well as their inappropriate disposal have led to widespread dissemination of antibiotics in various environments. Their abuse has resulted in a disproportionate rise in antimicrobial-resistant bacteria, which causes serious clinical infections, requiring aggressive therapy. Therefore, legislation regarding the control of antibiotics residues in live animals, animal products and environment is given in Council Directive 96/23/EC and maximum residue limits have been established for different matrices. So, it is critical to develop sensitive and selective analytical methods for regular monitoring and routine analysis of antibiotic in foods, clinical samples and the environment to ensure human and animal health.

In recent years, great efforts have been made to introduce high-throughput methods, so different methods have been developed. A general overview on the progress of the mains screening approaches to detect antibiotics residues including immunological, microbiological assays, classical chemical and biosensors is discussed. Some are being used routinely in diagnostic laboratories while others are still as research tools being used by academicians and specialists, which are in different stages of development. What are the pros and cons of using these different tools? Is one approach better than the other?

# Meeting report: O.1.2. COMPARATIVE STUDY FOR SOME ANTIOBESITY SUPPLEMENTS –STUDY FOR PERSONALIZED TREATMENT

**Authors:** B. Virgolici, M. Z. Constantinescu, D. Comandasu, D. Lixandru, D. Miricescu, E. Gubceag, L. Popescu, H. Virgolici, M. Mohora

**Affiliations:** 1 ”Carol Davila” University of Medicine and Pharmacy, Bucharest, Romania; 2 University of Agricultural Sciences and Veterinary Medicine, Bucharest, Romania

Many supplements are available for obesity treatment. The question is which supplement is most suitable in order to get the best management of obesity.

The aim of this experimental study was to do a comparison of the effects of three different antiobesity supplements on the obesity comorbidities, to get a personalized treatment.

Female Wistar rats were raised 15 weeks on a high caloric fat diet but in the last 6 weeks they were divided in groups according to the supplement which was associated with the fat diet. Group N took Nigella Sativa oil 0.1g/kg, group O took Omega 3 fatty acids 0.1g/kg, group F took Sea Buckthorn fruit 1g/kg, group HC didn’t take any supplements and group S changed from fat diet to standard diet in the last six weeks. In serum, the lipid profile, glycaemia and creatinine were measured. Histopathological examinations of liver, kidney, muscle and heart were done by hematoxylin-eozyin staining.

From different antiobesity supplements, when dyslipidemia and fatty liver dominate the obesity clinical picture, supplements rich in Omega-3 fatty acids are recommended. If dysglycemia and kidney and muscle dysfunction dominate the obesity clinical picture, the berry fruit supplements are recommended.

# Meeting report: O.1.3. STUDIES OF SOLVATED IONS IN CONFINED SPACES

**Authors:** F.D. Munteanu, A. Cavaco-Paulo, V. Beiu

**Affiliations:** 1 Aurel Vlaicu University, Arad, Romania; 2 Minho University, Campus de Gualtar, Braga, Portugal

It is well known that the neuronal membrane is covered with ion channels. Recent studies have shown that the distribution of ion channels is extremely regular on axons as well as on dendrites. Moreover, these ion channels are controlling the movement of ions across the neuronal membrane. As a consequence, the analysis of this very regular structures (regular networks) is of interest. For a better understanding of the neuron is necessary to understand the role of water as a solvent in biological systems. 

The present study is going to contribute to a better understanding of the neuronal system by taking into account the movement of the solvated ions in this confined space. 

*This work was funded by European Union through European Regional Development Funds Structural Operational Program “Competitiveness” Priority axis 1, Operation 1.1.4. Contract Number 30/2016*.

# Meeting report: O.1.4. INFORMATION AND COMMUNICATION TECHNOLOGY’S INFLUENCE ON APPROACHING MUSIC THERAPY

**Authors:** D. Drăgulin, F.A. Constantin

**Affiliations:** 1 Transilvania University of Braşov, Faculty of Music, Romania

There is no-doubt about the contribution Information and Communication Technology (ITC) has on favoring general access to education, to quality and efficient learning and teaching as well as on improving professional development and research. The practice of telemedicine, the internet-search self-diagnosis and, lately, the new experiment regarding the web-based symptom checkers give help and guidance to patients and are used in daily basis. Moreover, video conference callings between doctors and patients regarding a certain treatment, a primary diagnosis, blood test results interpretation or check–up cases are already in the medical practice.

Thus, the aim of this paper is to present a method of using ITC in the music therapy practice. We propose a guide that helps music therapists and practitioners to use ITC tools (video conferences, websites, soft programs, so on) in their daily activities to either offer therapeutic support, to obtain information about the clients, to have supervisors helping in a session, or to form a music therapy group or individual sessions with combined medical and therapeutic specialists. And although, the paper is a survey to help us start to experiment the method and less a conclusion based on real, measurable results, two things are true and verified: the existence of diverse technology applications in music therapy and the identified need of using ITC in current music therapy practice.

# Meeting report: O.1.5. MINIMAL RESIDUAL DISEASE ASSESSED BY MULTIPARAMETER FLOW CYTOMETRY IN ALL: THE PROGNOSTIC VALUE AT THE END OF INDUCTION CHEMOTHERAPY

**Authors:** C. Popa, O. Grigoraş, A. Colita, V. Atanasiu, D. Coriu

**Affiliations:** 1 Carol Davila University of Medicine and Pharmacy, Bucharest, Romania; 2 Fundeni Clinical Institute, Bucharest, Romania

The most frequent hematologic malignant disease in pediatric patient is acute lymphoblastic leukemia (ALL). The rate of blast clearance during therapy is a major prognostic factor of outcome in children with ALL. Blastic population can be recognized by their clonal rearrangement of immunoglobulin and T-cell receptor genes expression of gene fusions (using quantitative PCR) and leukemia-associated immunophenotypes (with multiparameter flow cytometry). These methods can detect one ALL cell among 10,000 to 100,000 normal cells in clinical samples. Owing these technology, minimal residual disease (MRD) is gaining clinical importance to establish: (1) the levels of MRD that are relevant to the therapeutic decision, (2) the relapse risk estimation that is generally proportional to the level of MRD when measured during or at the end of remission-induction therapy, (3) the clinical relevance of information that each method provides at the different time points. This is a review presentation on flow cytometry methodology and its contribution to MRD detection.

# Meeting report: P.1.1. CERVICAL CANCER CHEMOPREVENTION AND NATURAL POLYPHENOLS

**Authors:** M. Moga, O. Dimienescu, S. Casap, N. Bigiu, C. Arvatescu

**Affiliations:** * Transilvania University of Brasov, Faculty of Medicine, Brasov, Romania

There are many studies on human and animal cervical cancer cells that proved that polyphenols and their derivatives have antioxidant and anticancer potential. In the last years, the potential chemopreventive and chemotherapy properties of diet-derived agents have raised great interest among researchers. Despite the promising in vitro study results, clinical trials focused on the anticarcinogenic effects of polyphenols are missing. From the studies reviewed in this paper, we concluded that dietary polyphenols hold potential in the prevention and therapy of cervical cancer because they interfere in carcinogenesis (in the initiation, development and progression) by modulating the critical processes of cellular proliferation, differentiation, apoptosis, angiogenesis and metastasis. Specifically, polyphenols inhibit the proliferation of HPV cells, through induction of apoptosis, growth arrest, inhibition of DNA synthesis and modulation of signal transduction pathways.

# Meeting report: P.1.2. NEW ANALYTICAL METHODS FOR ALLANTOIN DETECTION

**Authors:** M. Balan, S. Chiperea, L. Floroian, L. Gaceu, M. Badea

**Affiliations:** 1 Transilvania University of Brasov, Faculty of Medicine, Romania; 2 Transilvania University of Brasov, Faculty of Electrical Engineering and Computer Sciences, Romania; 3 Transilvania University of Brasov, Faculty of Food and Tourism, Romania

Allantoin is considered to be the end product of purine metabolism in most mammals, but not in humans. A marked attention has been drawn to the idea that allantoin might derive from the transformation of uric acid in the presence of oxidative species. This idea is very intriguing and has led to many works in which allantoin is proposed as marker of illnesses in which oxidative stress plays a major role.

The aim of this study was to propose new methods for the detection of allantoin, with a main focus on electrochemical and spectrophotometric methods, as possible alternative for chromatographic methods mainly reported in scientific literature.

We have studied the electrochemical detection of allantoin through Differential Pulse Voltammetry. For a better optimization, we analyzed different electrodes, noted the influence of pH and have taken uric acid as a possible interference substance. 

Allantoin was analysed using UV spectrometry, using the same conditions as those in the electrochemical analysis. It was found a specific peak for allantoin to be around the value of 218 nm, slightly influenced by pH but with no interference from uric acid.

Similar studies on real probes (extracts of allantoin from plants) were done with good results.

The analytical experiments in this study show two promising directions of allantoin detection, with a further need for more research in optimizing detection of real biological samples, including biological ones, which could eventually prove of value in clinical trials involving diseases caused by oxidative stress.

# Meeting report: P.1.3. PREVALENCE OF RISK FACTORS FOR THE CARDIOVASCULAR DISEASES IN RELATION TO AGE

**Authors:** E. Lupeanu, R. Hnidei, I. Răducanu, B. Moroşanu, O.N. Peneş, P. Gherasim, D. Roditis

**Affiliations:** * National Institute of Gerontology and Geriatrics „Ana Aslan”, Bucharest, Romania

Metabolic changes and increased blood pressure in aging may become risk factors for cardiovascular diseases (CVD) associated with aging process.

The aim is to assess metabolic parameters and blood pressure and to evidence the prevalence of the risk factors for cardiovascular events related to age.

Patients were divided into groups according to age: **A**: 50 - 59 years; **B**:60 - 69 years; **C**:70 - 79 years; **D**:80 - 92 years. We measured the serum levels for glucose, urea, creatinine, uric acid, total cholesterol, HDL-cholesterol, non-HDL-cholesterol, LDL-cholesterol, triglycerides, transaminases and total proteins and we calculated the ratios: total cholesterol/HDLc, LDLc/HDLc, non-HDLc/HDLc, triglycerides/HDLc and blood pressure.

Investigations showed significant increase in creatinine (p<0.025), urea (p<0.001) and uric acid (p<0.0205), and significantly decrease in HDL cholesterol (p<0.022) with age. Glucose levels (>110 mg/dL), total cholesterol (> 220 mg/dl), LDL cholesterol (>130 mg/dL) and triglycerides (>150 mg/dL) and values of HDL cholesterol <40 mg/dL in men and <50 mg /dl in women are metabolic risk factors for CVD. 25-56% of patients of all ages are at risk for CVD. All age groups have patients with increased atherogenic risk (col/HDL>5 and LDL/HDL>3.5). Also, there was a significant increase of systolic blood pressure in patients aged 60-69 years (p = 0.0013) and 70-79 years (p = 0.0007). Adults, 45-59 years, have the lowest prevalence of hypertension. 

The study showed metabolic and blood pressure changes with age and pointed out the risk factors for cardiovascular diseases associated with the aging process.

*Studies were done under contract nr. 335/2014 (PN II PCCA 2013 -4-1686)*.

# Meeting report: P.1.4. COMPREHENSIVE GERIATRIC ASSESSMENT ROLE IN EXTENDING ACTIVE LIFE FOR INDEPENDENCE AND HEALTHY AGING

**Authors:** E. Lupeanu, D. Roditis

**Affiliations:** * National Institute of Gerontology and Geriatrics „Ana Aslan”, Bucharest, Romania

Clinical trials have shown that elderly patients have a multiple for which they consume more drugs than adults.

The aim is to point out the health profile of the elderly and to identify risk factors for diseases associated with aging. The objective is the comprehensive geriatric evaluation of subjects of different ages, in order to set off bio-medical and socio-economic factors, the eating habits and lifestyle that may have an important role in modulating aging, lifespan and quality of life of the elderly. 

Comprehensive geriatric assessment included clinical examination, laboratory, functional (ADL, IADL), metabolic, nutritional and cognitive (MMSE) evaluations, the practices related to health and lifestyle: smoking, alcohol consumption, eating habits, socio-economic conditions of patients included in the study. There were investigated 250 subjects divided into age groups: **A**: 45-59 years, **B**: 60-69 years, **C**: 70-79 years and **D**: 80-90 years. 

Complex geriatric assessment showed the physical, metabolic, functional and cognitive changes with patients age, which have led to the development of self-assessment tests of health and lifestyle. They may be the consequence of lifestyle and therapeutic strategies adopted by subjects, may constitute risk factors for various diseases associated with the aging process and together define the patient health status. 

This type of assessment allows a personalized intervention, such as: rehabilitation, education, counseling, supportive services. 

Comprehensive geriatric assessment may have some benefits: higher accuracy of diagnosis, clinical care according to results, improved functional and mental status, mortality reduction and, consequently, reduced acute care hospitals. 

***Acknowledgments:** Studies were done under contract nr. 335/2014 (PN II PCCA 2013 -4-1686)*.

# Meeting report: P.1.5. OBESITY: LINK BETWEEN ADIPONECTIN AND CARDIOVASCULAR DISEASE

**Authors:** T. Holban, L. Gaman, I. Stoian, V. Atanasiu, C. Muscurel

**Affiliations:** 1 Medical Laboratory, Tandarei Hospital, Tandarei, Romania; 2 ”Carol Davila” University of Medicine and Pharmacy, Biochemistry Dept, Bucharest, Romania

Obesity, especially visceral obesity is associated with chronic inflammation and an increased risk of cardiovascular diseases.

The purpose of this study is to investigate any correlations between adiponectin, IL-6, triglycerides and HDL-cholesterol in obese and non-obese subjects.

 The study included 20 obese subjects (all men) with BMI ≥ 30 (body mass index) and 20 normal subjects (all men and the same age). We have determined the serum levels of adiponectin (ELISA), IL-6 (chemiluminescence), triglycerides and HDL-cholesterol (spectrophotometry).

Obese patients presented significantly lower serum adiponectin than normal subjects and this is negatively correlated with IL-6 (p < 0, 05) and triglycerides (p < 0,05) and positively correlated with HDL-cholesterol. Experimental findings have shown that adiponectin protects the cardiovascular tissues under conditions of stress related to the stimulation of endothelial cell responses and the inhibition of proinflammatory and hypertrophic responses.

Hypoadiponectinemia is a marker of cardiovascular disease. The finding of pharmacologic agents able to improve adiponectin plasma levels should be target of future research.

# Meeting report: P.1.6. HEPATIC STEATOSIS-ASSOCIATION BETWEEN BIOCHEMICAL FEATURES AND CONTROLLED ATTENUATION PARAMETER

**Authors:** G.C. Robu, L. Gaman, V. Atanasiu, I. Stoian, D.V. Balaban, F. Ionita-Radu, R.S. Costache, P. Nuta, S. Bucurica, A. Popescu, B. Macadon, M. Patrasescu, D. Gheorghe, M.C. Robu, M. Jinga

**Affiliations:** 1 Carol Davila Central Military Emergency University Hospital, Bucharest, Romania; 2 Carol Davila University of Medicine and Pharmacy, Bucharest, Romania; 3 University of Medicine Titu Maiorescu, Bucharest, Romania

Controlled attenuation parameter (CAP) using transient elastography (Fibroscan) is a recent non-invasive tool for assessment of steatosis.

Our aim was to evaluate steatosis in patients with chronic liver diseases using CAP. We included 49 patients (26 female and 23 male, mean age 62 years old) with chronic liver diseases (hepatitis B, hepatitis C, cirrhosis, steatohepatitis and primary biliary cholangites). 

All patients were investigated using the 3,5MHZ standard M probe. Hepatic steatosis (any grade involving more thean 10% of hepatocytes) was defined as CAP 230 dB/m. We analyzed the relationship between CAP and the following clinico-biological parameters: age, gender, liver stiffnes, level of cholesterol and tryglicerides.

Steatosis (CAP) was predominantly associated with hepatitis C in 46 % of patients.A higher value of CAP was also seen in patients with cirrhosis (35%).We also noticed an important association between steatosis and hypercolesterolemia in 60% patients and with hypertriglyceridemia in 100% patients.In contrast, a normal value of CAP was associated with a higher value of liver stiffnes (in 57% patients ).

CAP offers an immediate assessment of steatosis. The accurate assessment of liver steatosis is crucial in clinical practice for the management of patients with chronic liver diseases and in clinical research.

# Meeting report: P.1.7. MODERN STUDIES FOR ELECTROCHEMICAL DETECTION OF DOPAMINE, SEROTONIN AND ASCORBIC ACID

**Authors:** S. Chiperea, M. Balan, L. Floroian, M. Badea

**Affiliations:** 1 Transilvania University of Brasov, Romania; 2 Transilvania University of Brasov, Faculty of Electrical Engineering and Computer Sciences, Brasov, Romania

Because of the important roles of dopamine, serotonin and ascorbic acid in a series of illnesses or physiological events inside the human bodies, individual measurements of these compounds could represent the future of diagnosis and follow up of these diseases. 

Due to its high sensitivity, high reproducibility rate and low costs, differential pulse voltammetry (DPV) was chosen as the optimum electrochemical method for the detection of dopamine, serotonin and ascorbic acid, using a PalmSens potentiostat and glassy carbon sensors connected to the PSTrace software. The experiment was performed using three pH values (5.8, 7 and 7.4 – phosphate buffer), in which increasingly volumes of the three compounds were added. Each substance was analyzed (single and in combination with other compounds) at each pH value. The data we extracted using the PSTrace software regarded the height and area of each specific peak for every compound and the potential at which this peak appeared. Calibration curves were performed.

Based on the above results, DPV could be recommended for testing real samples (blood, saliva, urine) in order to detect these compounds.

# Meeting report: P.1.8. CHARACTERISTIC FEATURES OF CHRONIC LYMPHOCYTIC LEUKEMIA - A RETROSPECTIVE STUDY ON 39 PATIENTS FROM FUNDENI CLINICAL INSTITUTE

**Authors:** A Chivi, O. Grigoraş, G. Iosub, P. Soare, P. Buciu, V. Atanasiu, L. Gaman, C. Popa C

**Affiliations:** 1 Carol Davila University of Medicine and Pharmacy, Bucharest, Romania; 2 Fundeni Clinical Institute, Bucharest, Romania

Chronic lymphocytic leukemia (CLL) is a monoclonal disorder characterized by progressive accumulation of CD5-positive lymphocytes in peripheral blood, bone marrow, lymph nodes and spleen. The Binet staging system categorizes patients according to the number of lymph node groups involved, hemoglobin, number of platelets and clinical prognostic features of the patients.

The main objective of the study was to identify some correlations, at the time of diagnostic, between clinical characteristics (Binet stage) and age, gender, some laboratory parameters and some well-known prognostic factors (CD38, β2- microglobulin).

For 39 patients (average age 64) who had been diagnosed with CLL in Fundeni Clinical Institute, from 1st January 2016 to 31st December 2016, Binet staging, blood count, CD38 and β2- microglobulin were analysed.

The study showed that the male dominance was evident with a ratio male/female = 1.78. Also, statistical associations between gender and Binet Stage were noticed. The number of lymphocytes count was significantly more increased in patients with stage C rather than in the group with stages A or B (p=0.0047). The prognosis marker CD38 was positive only in 5 patients, 4 of them being in stage A or B. No statistically significant differences in the levels of β2- microglobulin between the different stages of Binet Classification were found.

This study defined some characteristics of patients with CLL: the patient type would be male, age between 55-70, in stage A or B according to Binet stage, with very high lymphocyte count, no positivity for CD38 that could be a predictive marker for a shorter overall survival rates.

# Meeting report: P.1.9. DYNAMIC STUDY ON CARBAPENEM RESISTANCE OF GRAM NEGATIVE BACILLI

**Authors:** M. Idomir, M. Badea

**Affiliations:** * Transilvania University of Brasov, Brasov, Romania

Resistance to antibiotics currently represents globally a priority issue of public health. The last decades are characterized by the rapid increase in the number of multi-resistant antibiotic strains in clinical isolates and the reduced rate of introduction of new antimicrobial drugs in therapy. Carbapenems are considered the drugs of last resort for infections with multi-resistant gram negative bacilli. The selection of resistant strains to these antibiotics is a serious threat, resulting in high rates of morbidity and mortality through infections similar to those prior to the antibiotic era.

The purpose of our study was to evaluate the resistance to carbapenems of Gram negative bacilli isolated from various samples of patients admitted to a multidisciplinary clinical hospital over a period of 2 years (1.01.2015-31.12.2016). The results obtained revealed high levels of resistance to imipenem in the case of *Acinetobacter species* (75.55%) and *Pseudomonas aeruginosa* (35.06%). With very low levels of resistance were also detected strains of *Enterobacteriaceae resistant* to imipenem (*Serratia* spp. - 2.77%, *Klebsiella* spp. - 1.36%, *Proteus spp.* - 0.81%, *E. coli* - 0.15%).

The study demonstrated the presence of carbapemenase-producing bacterial strains in the samples of the studied patients as well as a tendency to increase their share from one year to the other in *Acinetobacter spp.*, *P. aeruginosa* and *Serratia spp*.

# Meeting report: P.1.10. SERUM BIOMARKERS IN PREDICTING LIVER DAMAGE AMONG CHRONIC HEPATITIS C INFECTED PATIENTS

**Authors:** R. Ilies, P. Buciu, A. Gherman, A. R. Barnea, A. O. Ion, A. L. Zamfir, A. Hristu, C. Muscurel, L. Gaman, C. Popa

**Affiliations:** 1 “Carol Davila” University of Medicine and Pharmacy, Bucharest, Romania; 2 Fundeni Clinical Institute, Bucharest, Romania

Worldwide, more than 185 million people live with Hepatitis C Virus (HCV) who remains the major cause of morbidity and mortality. 75%-85% of people who get infected with HCV develop chronic infection and 20% of them experience progression to liver cirrhosis, hepatocellular cancer, liver failure and death. Liver failure as a consequence of HCV infection is one of the most common reasons for liver transplantation. Liver biopsy is considered as the golden standard for staging, however, it is invasive and has risk of complication. Because of these limitations, numerous studies have focused on developing simple non-invasive biochemical markers as an alternative to liver biopsy.

The objective of this study was to evaluate and determine the potential correlation between liver fibrosis assessed by liver biopsy and different biochemical parameters in untreated chronic hepatitis C (CHC) infected patients. We performed a retrospective study on 46 untreated CHC patients. The diagnosis was established by the presence of HCV antibody on electrochemiluminescence assay and confirmed by the presence of HCV RNA using real-time PCR. Complete blood counts, 24 biochemical parameters [gamma-glutamyltranspeptidase (GGT), aspartate aminotransferase (AST), alanine aminotransferase (ALT), amylase, lipase, direct bilirubin (DBIL), total bilirubin (TBIL), creatinine (CREA),urea (UREA), cholesterol (CHOL), high density lipoprotein (HDL), low density lipoprotein (LDL), very low density lipoprotein (VLDL), triglyceride (TRIG), transferrin, ferritin, total iron-binding capacity (TIBC), serum iron, glycaemia (GLU), alpha-fetoprotein (AFP), alkaline phosphatase (ALP), sodium (Na+), chlorine (Cl-), potassium (K+)], were measured with automated analyzer. Liver biopsy was recorded for each patient. 

31 females and 15 males with a mean age of 50±12.2 years at beginning of antiviral therapy were included in the study. No statistical association bettween biochemical parameters and HCV viral load was found. We used a random forest classification algorithm in order to obtain a model with the most important variables that predict severe fibrosis, the decreasing importance of these parameters being: TBIL, GGT, AST, K+, hemoglobin (Hb), serum iron, AFP, TCHOL, transferrin. 

Among CHC patients, hemoglobin and eight biochemical parameters can be predictor factors for progressive liver disease.

# Meeting report: P.1.11. THE EVALUATION OF THE INCIDENCE AND CHARACTERISTICS OF IUGR IN THE CLINICAL HOSPITAL OF OBSTETRICS AND GYNECOLOGY- BETWEEN 2010-2014

**Authors:** D. Panait, A. Maxim, C. Martinescu, A. Maier, N. Bigiu, M. Moga

**Affiliations:** 1 Transilvania University of Brasov, Romania; 2 Clinical Emergency County Hospital Brasov, Romania

After prematurity, intrauterine growth restriction (IUGR) represent the second cause of perinatal mortality. Newborns with IUGR have a 5 up 10 times higher tisk of intrauterine death with a chance of delivery between 23-65%. Almost half of the stillbirths present prematurity and a ¼ of the term stillbirths present IUGR. Our study is a retrospective study developed on a 5 years period on the births registered at the Clinical hospital of obstetrics and gynecology „Dr. I.A. Sbarcea”, Brasov of which 241 meet the strict diagnose of IUGR. We have settled correlations to the maternal age, none of the cases diagnosed with IUGR being connected to an older mother. In the study group, 98 newborns have a weight under 2500g at birth, the remaining 143 weighing more than 2500g; related to the newborn male: female ratio we have noticed that IUGR has a high frequency among male newborns: 58,5%- result contrary to the scientific literature. The environmental origin of the mother represent a risk factor for the development of IUGR due to multiple causes. In our study group, 137 cases of pregnancies presenting IUGR live in the countryside.

The conclusion is that our study has identified local characteristics of the patients presenting with IUGR pregnancies that can further precocious orient the doctor towards a possible development of such a pathology

# Meeting report: P.1.12. MICROWAVE OVENS EMIT ELECTROMAGNETIC FIELD: STUDY OF THE RADIATION LEAKAGE

**Authors:** D. Panait, A. Jufa, L. Floroian, A. Pascu, M. Badea

**Affiliations:** 1 Transilvania University of Brasov, Faculty of Medicine, Brasov, Romania; 2 Transilvania University of Brasov, Faculty of Electrical Engineering and Computer Sciences, Brasov, Romania

Microwaves are electromagnetic waves with higher frequencies than those of radio waves and lower than those of infrared band. Nowadays the microwave ovens are widely used due to their short time needed for warming foods. We studied the electromagnetic fields that microwave ovens available for domestic use emit and compared the results to the scientific literature.

Our results show radiation leakage levels higher than the allowed levels recognized by different scientific sources in front of the microwave ovens. Due to our results we strongly recommend limitation of the usage of such devices and highlight marked need of further investigation in this field.

# Meeting report: P.1.13. OPPORTUNITIES IN TELEMEDICINE - AN ETHICAL POINT OF VIEW

**Authors:** F. Leasu, N. Dinu, A. Repanovici, M. Badea, L. Rogozea

**Affiliations:** * Transilvania University of Brasov, Faculty of Medicine, Brasov, Romania

Telemedicine has become in recent years an increasingly invoked way in medical systems because of the lack of medical professionals in inaccessible or less attractive areas. The graduates from faculties of medicine or study programs as physiotherapy or medical clinical laboratory are not eager to work in this areas.

In this context it becomes increasingly difficult to balance the role of telemedicine, and establishing a demarcation between technology, efficiency and reducing costs and keeping medicine humanistic is a continuous challenge. Linking national networks to the international ones is not just a way to generalize the information obtained, very useful especially for rare diseases, as well as a way to benefit from the experience of other professionals, according to confidentiality of data obtained. 

In conclusion, telemedicine can be a way to accelerate the reforms in the healthcare system, but also an opportunity to develop the medical education on other principles.

# Meeting report: P.1.14. GLOBAL VERSUS LOCAL IN E-MEDICINE AN ETHICAL PERSPECTIVES

**Authors:** L. Rogozea, N. Andrea, T. Lavinia, D. Popa

**Affiliations:** 1 Transilvania University of Brasov, Faculty of Medicine, Brasov, Romania

The development of e-medicine is an opportunity for both medical staff and medical educators.

Global health is a term becoming more used worldwide, becoming fashionable to use it and turning it into an absolute priority. However global development can not cover all health problems and local development becomes as much important for local authorities in any country.

In this context the role of health educators is to introduce in the curricula of any doctor, both during basic training, as well as the internship, topics of bioethics which have to be correlated both with global health and local health problems.

Development of best practice guidelines in the field of ethics similar to those we have in other medical specialties is an unattainable goal yet, but possible to achieve in the coming years. But for these guides to appear it is absolutely necessary to change optics and to transform ethics in a clear concern especially for medical professionals; in this way medical ethics will become much less abstract and inclined towards philosophy but more integrated with practice.

# Meeting report: P.1.15. TWO DECADES OF EXPERIENCE WITH THE STUDY PROGRAM HEALTHCARE BIOANALYTICS AT THE CHARLES UNIVERSITY FACULTY OF PHARMACY

**Authors:** J. Dršata

**Affiliations:** * Charles University Faculty of Pharmacy in Hradec Králové, Hradec Králové, Czech Republic

Education of *Laboratory technicians and Specialists in laboratory methods* is organized in the Czech Republic according to the act No. 96/2004, which defines conditions for their erudition. Example of a school that offers such education is the Charles University Faculty of Pharmacy. Besides the five-year Pharmacy Master study course, also the program of Healthcare Bioanalytics is taught at the faculty. In accord with recommendation of Bologna declaration, the program comprises of two undergraduate steps – a three-year Bachelor program, and a linking-up two-year Master program. The graduate from the Master studies may continue her/his education in doctoral studies (PhD).

The graduate from Bachelor studies is a university-trained healthcare professional capable of working in all types of clinical and sanitary service laboratories at the position of Laboratory technician. The graduate from the Master program is trained for future independent work and management activities in all types of clinical laboratories and sanitary service at the position of Specialist in laboratory methods.

The three-step studies in Hradec Králové represent the only complete accredited program for education of non-medical healthcare laboratory specialists in the Czech Republic. The graduate is prepared to be advisor to physicians and to laboratory co-workers in laboratory methods, in the clinical relevance of laboratory results and in their interpretation, or to enter a scientific career in biomedical sciences. 

A survey of the current curriculum of the program and experience with two decades of its development will be discussed. 

# Meeting report: P.1.6 COMPARATIVE STUDY REGARDING THE KNOWLEDGE LEVEL ABOUT URIC ACID AND ELECTROCHEMICAL METHODS FOR ITS DETECTION

**Authors:** M. Pirvu, L. Floroian, M. Badea

**Affiliations:** 1 Transilvania University of Brasov, Faculty of Medicine, Brasov, Romania; 2 Transilvania University of Brasov, Faculty of Electrical Engineering and Computer Sciences, Brasov, Romania

Uric acid is a white crystal represeting the end product of protein metabolism. It is found in the most part in blood and urine, when found in high concentrations in the body it begins to form uric acid crystals. Besides the implication in various conditions this heterocyclic compound has positive effects on the human body by possesing the power of destroying the harmful radicals. Uric acid it is in charge with more than a half of the antioxydant capacity in plasma. Recent studies was shown that latetly it has increased the involvement in numerous conditions such as cardiovascular disease, cancer and diabetes and it has a much higher and negative impact in the develpement of the illnesses when associated with them. 

The aim of this work were to analyse the level of knowledge concerning uric acid (its role in the human body – effects and protection) and to develop and to optimize different electrochemical detection methods for uric acid detection. We have conducted a comparative questionnaire study amoung the medical students in the fourth and the final year from the Faculty of Medicine, Transilvania University of Brașov, Romania in order to test the level of informaton about uric acid aquired during the years of study. Relating to the laboratory study, we have tested Differential Pulse Votametry as possible method for detection of uric acid, using different buffer systems and different electrods.

Analysing obtained results it was demonstrated that the level of students knowledge are in direct dependence by the level of their study (preclinical and clinical years), and future plans for their improving are necessary. Regarding the uric acid electrochemical determination it showed a high sensitive at pH 7, result that is in according to the speciality studies.

# Meeting report: P.1.17. BRAIN ACCESSIBILITY AND NEUROPROTECTION OF SELECTED FLAVONOIDS

**Authors:** L. Pogačnik, K. Pirc, I. Palmela, M. Skrt, D. Brites, M. A. Brito, N. Poklar Ulrih, R. F. M. Silva

**Affiliations:** 1 Biotechnical Faculty, Department of Food Science and Technology, Ljubljana, Slovenia; 2 Research Institute for Medicine and Department of Biochemistry and Human Biology, Universidade de Lisboa, Lisbon, Portugal; 3 Centre of Excellence for Integrated Approaches in Chemistry and Biology of Proteins, Ljubljana, Slovenia

The projections for a great intensification in the number of neurodegenerative disease’s cases due to the increase in life expectancy outlines the importance of protective and therapeutic strategies to circumvent neurodegeneration. Natural food sources may constitute a promising font for new compounds with neuroprotective properties once they are able to reach the brain. As so, our aim was to evaluate the brain accessibility and neuroprotection abilities of quercetin (Q), epigallocatechin gallate (EGCG) and cyanidin-3-glucoside (C3G). Primary cortical neuron cultures and an optimized human blood-brain barrier (BBB) model were used. Although it seems that Q does not reach the “brain side” of our BBB model, this is crossed fast by EGCG and slowly by C3G. We found that EGCG was the most effective among selected compounds, reducing oxidative-induced neuronal necrotic-like cell death by ~40% and apoptosis by ~30%. At the same time, all of them effectively inhibited α-synuclein fibrillation over the relevant timescale applied here. Finally, EGCG revealed to be the most promising with both the capability to reach the brain and to protect neurons from oxidative damage.

*Work supported by Fundação para a Ciência e a Tecnologia (FCT), Portugal, (UID/DTP/04138/2013) and Slovenian Research Agency (research core funding No. P4-0121)*.

# Meeting report: P.1.18. INTERACTION STUDY OF LACTOFEN WITH DNA AND VOLTAMMETRIC DETERMINATION OF DNA IN AQUEOUS SOLUTIONS

**Authors:** S. Smarzewska, T. Popławski, K. Morawska, D. Guziejewski, W. Ciesielski

**Affiliations:** 1 Department of Inorganic and Analytical Chemistry, Faculty of Chemistry, University of Lodz, Lodz, Poland; 2 Department of Molecular Genetics, University of Lodz, Lodz, Poland

Lactofen (LCT, Ethyl O-[5-(2-chloro-α,α,α-trifluoro-p-tolyloxy)-2-nitrobenzoyl]-DL-lactate) is a selective herbicide used to control broadleaved weeds in soybean, cereals, potatoes and peanuts. 

Voltammetric determination of lactofen was performed using cyclic renewable silver amalgam electrode (Hg(Ag)FE) and square wave stripping voltammetry (SWSV). LCT electrochemical properties were studied in a broad range of Britton-Robinson (pH 2.0-10.0) and borate buffer (8.0-10.0). The highest signal was recorded in borate buffer pH 8.5 at the potential ca. -0.5 V. Optimal conditions were as follows: conditioning potential -1.0 V, conditioning time 5 s, SW frequency 50 Hz, SW amplitude 50 mV, step potential 7 mV, quiescent time 5 s, deposition potential and time -0.2 V and 150 s, respectively). Linear dependence of the recorded peak current and LCT concentration was observed in the concentration range from 5·10-8 to 2.5·10-7 mol·L-1. The detection and quantification limits were found to be 9·10-9 and 3·10-8 mol·L-1, respectively. Later, square wave and cyclic voltammetry at a Hg(Ag)FE electrode were used for the study of the interaction of lactofen with double-stranded DNA in PBS buffer pH 7.4. The kinetic parameters of the reduction of LCT and the DNA–LCT complex and the thermodynamic parameters of the formed DNA–LCT complex were calculated. Moreover, the calibration dependence of the peak current of LCT on the concentration of DNA in PBS buffer was constructed to be used for the indirect determination of DNA based on the decreasing peak current of LCT with increasing concentration of DNA in the measured solution.

# Meeting report: P.1.19. ELECTROCHEMICAL AND SPECTROSCOPIC STUDIES OF THE INTERACTION BETWEEN THE ANTIVIRAL DRUG TENOFOVIR AND DNA

**Authors:** S. Smarzewska, T. Popławski, K. Morawska, D. Guziejewski, W. Ciesielski

**Affiliations:** 1 Department of Inorganic and Analytical Chemistry, Faculty of Chemistry, University of Lodz, Lodz, Poland; 2 Department of Molecular Genetics, University of Lodz, Lodz, Poland

The interaction between DNA and other molecules is an important fundamental issue in life sciences which is related to the replication and transcription of DNA in vivo, mutation of genes and related variations of species in character, action mechanisms of DNA-targeted drugs, origins of diseases, and action mechanisms of some synthetic chemical nucleases. Tenofovir (TFV; 1-(6-aminopurin-9-yl) propan-2-yloxymethylphosphonic acid) belongs to nucleoside reverse transcriptase inhibitors used to treat chronic hepatitis B and to prevent and treat HIV/AIDS.

First, using boron doped diamond electrode simple and precise voltammetric method for quantitative determination of TFV in bulk form and pharmaceutical formulation was developed. It was shown that in analysis of real samples, direct analysis was possible and no time-consuming preparation steps were necessary. Next, the interaction between tenofovir and single/double stranded DNA was investigated with cyclic voltammetry and UV/vis spectrophotometry. 

# Meeting report: P.1.20. SQUARE-WAVE VOLTAMMETRIC RESPONSE IN ESTIMATION OF ELECTRODE PROCESSES KINETICS BASED ON AMPLITUDE ALTERATION

**Authors:** D. Guziejewski, S. Smarzewska, V. Mirceski

**Affiliations:** 1 Department of Inorganic and Analytical Chemistry, Faculty of Chemistry, University of Lodz, Poland; 2 Institute of Chemistry, Faculty of Natural Sciences and Mathematics, University “Ss. Cyril and Methodius”, Skopje, Republic of Macedonia

Most of electrochemical methods of electrode reactions kinetics determination are based on studying the effect of time scale of the experiment, i.e. scan rate (in cyclic voltammetry) or frequency (in square wave voltammetry) on the recorded signal (peak current, potential, half peak width and/or its combination). Recently the splitting of the net response in SWV with alteration of amplitude was pronounced with its effect on overall rate of electron transfer. Here we report the employment of amplitude effect on the amplitude-normalized SW net peak current as well as on the SW component peak potential separation. Our first studies were related with establishing the basic methodology in the case of a simple solution-phase electrode reaction at a planar or spherical electrode, a solution phase electrode reaction coupled with a reversible follow-up chemical reaction and a diffusion less surface confined electrode reaction. The experimental studies were confirmed with studying kinetics of 2-methyl-2-nitropropane as well as azobenzene and hexacyanoferrate systems. Here we would like to report the application of the developed method for other biologically important compounds. Simple experimental setup can be easily achieved and exploited for electrode reaction kinetics determination at a fixed frequency. Electrode reaction kinetics is assessed based on regular SW components peak potential separation as well as on amplitude-corrected SW components peak potential separation. Electrode reaction standard rate constant can be derived using particular working equation of user-defined electrode mechanism or with direct fitting of theoretical simulation with experimental results. *We acknowledge with gratitude the support from the National Science Centre of Poland through the grant 2011/03/N/ST4/01338*.

# Meeting report: P.1.21 INTERACTIONS OF PROFLURALIN WITH DNA ANALYZED WITH ELECTROCHEMICAL METHODS

**Authors:** D. Guziejewski, S. Smarzewska, T. Popławski, W. Ciesielski

**Affiliations:** 1 Department of Inorganic and Analytical Chemistry, Faculty of Chemistry, University of Lodz, Poland; 2 Department of Molecular Genetics, University of Lodz, Poland

Herbicides are chemical substances used to control weeds. The analysis of herbicides is an important field of analytical chemistry undergoing rapid development. These compounds protect agricultural crops, but overuse and incorrect use can pose risks to human health and the environment. An example of selective herbicide is profluralin (N-(cyclopropylmethyl)-2,6-dinitro-N-propyl-4-(trifluoromethyl)aniline, PFL), a compound belonging to the group of plant growth regulators. PFL is used for inhibiting the growth of grasses and broadleaf weeds common for cabbage, tomato, cotton, sunflower, peanuts, beans and peas cultivation. PFL penetrates weed’s roots and stems, inhibiting their growth in germination stage.

Voltammetric determination of profluralin was performed using square wave voltammetry (SWV) along with stripping stage (SWSV). PFL electrochemical properties were studied in a broad range of Britton-Robinson, borate and borax buffer. The signal was analysed with addition of ethanol as well as surface active compounds. Optimal conditions were as follows: frequency 80 Hz, amplitude 30 mV, step potential 5 mV. Linear dependence of the recorded peak current and PFL concentration was observed in the concentration range from 4·10-7 to 7·10-6 mol·L-1. When accumulation stage is involved with optimal deposition potential and time -0.1 V and 60 s, respectively the linear concentration range was found to be from 6·10-8 to 8·10-7 mol·L-1. The determination method was applied to the analysis of spiked environmental samples like tap water, Ner river water and soil. Later, voltammetric techniques with Hg(Ag)FE electrode were used for the study of the interaction of profluralin with DNA.

# Meeting report: P.1.22. SELF-PERCEPTION OF QUALITY OF LIFE FOR DYALIZED PEOPLE

**Authors:** O. Barbat, L. Rogozea, D. Panait, M. Badea

**Affiliations:** * Transilvania University of Brasov, Faculty of Medicine, Romania

Chronic kidney disease is the subject of intense debate in specialist nephrology studies due to the threshold glomerular filtration rate threshold used to diagnose the disease. We conducted a multicenter comparative study between March 2016 and August 2016, based on the Kidney Disease and Quality of Life ™ Short Form 13 (KDQOL-SF ™) questionnaire conducted by RAND and the University of Arizona.

The questionnaire was validated in Romania by the Romanian Society of Nephrology in 2008.

The study group consisted of a total of 256 patients, of whom 200 performed hemodialysis and 56 peritoneal dialysis from 01.03.2016 to 17.08.2016.

The main concern of this study was to examine the quality of life of patients undergoing hemodialysis compared to those of peritoneal dialysis.

The results of the study provide evidence that hemodialysis patients have achieved better outcomes in areas such as daily activities, physical activities, social activities, sexual activity, sleep, and mental health. Regarding the impact of renal disease, equal scores between hemodialysis patients and peritoneal dialysis were seen to have a negative impact on their life.

# Meeting report: P.1.23. HUMAN EXPOSURE TO VIBRATION AND OCCUPATIONAL NOISE AND ASSOCIATED DISSEASE

**Authors:** M. Badea, M. Radoi, L. Baltes, L. Floroian, M. Tierean, D. Senchetru

**Affiliations:** 1 Transilvania University of Brasov, Faculty of Medicine, Romania; 2 Transilvania University of Brasov, Faculty of Material Science and Engineering, Brasov, Romania; 3 Transilvania University of Brasov, Faculty of Electrical Engineering and Computer Sciences, Brasov, Romania; 4 Territorial Labor Inspectorate Brasov, Romania

Due to the effects of the noise on the world, a series of measures have been taken to reduce the noise level both in the environment and in the workplace.

Once Romania joins the EU, Romania has the obligation to implement and comply with the noise reduction measures.

The paper aim is to study comparatively the effects of noise on the body.

Results of studies in different work environments with different noise intensities were presented, as well as issues regarding the implementation of noise management.

Involvement of the employee and employer in established protection measures, as well as specialized examinations at workplaces, will reduce the possible noise effects.

# Meeting report: P.1.24. A PILOT STUDY REGARDING THE PROGNOSTIC SIGNIFICANCE OF SOME SERUM PARAMETERS IN THE DEVELOPMENT OF MICROALBUMINURIA AND DIABETIC NEPHROPATHY

**Authors:** P. Buciu, A. Botez, A. C. Bucur, N. Jigău, C. Mironică, A. Muraru, M. Petran, M. Badea, L. Gaman, C. Popa

**Affiliations:** 1 Carol Davila University of Medicine and Pharmacy, Bucharest, Romania; 2 Fundeni Clinical Institute, Bucharest, Romania; 3 Transilvania University of Brasov, Faculty of Medicine, Brasov, Romania

Diabetes mellitus, affect more than 346 million people worldwide, is characterized by hyperglycemia due to a deficiency of insulin that affects the metabolism of carbohydrate, protein, fat, water and electrolytes. Diabetic kidney disease (DKD) is the most common leading cause of chronic kidney disease and end‐stage renal disease (ESRD). The present study was to investigate whether the presence of microalbuminuria in patients is predicted by some common hematological and biochemical parameters, in order to improve understanding of the development and progression of DKD.

We performed a retrospective study on 97 patient, 52 females and 45 males (ages range between 4-82 years), with Diabetes Mellitus type II and microalbuminuria measured as albumin-to-creatinine ratio (ACR) in a first morning spot collection and Complete blood counts and eleven serum biochemical parameters (albumin, aspartate aminotransferase (AST), total bilirubin (TBIL), creatinine (CREA),urea (UREA), cholesterol (CHOL), glycemia (GLU), triglyceride (TRIG), sodium (Na+), total protein) were measured with automated analyzer. Using multivariate logistic regression on our dataset (all biochemical parameters, age and hemoglobin), we obtain, by cross-validation, a five-variable equation which can be used to calculate the probability that the patient has microalbuminuria, hence it may quantify the risk of progression to chronic renal disease. 

After applying our statistical model, we observed that a particular model composed of age and other 4 serum parameters (albumin, total bilirubin, creatinine and hemoglobin), had the best predictive performance as measured by repeated 10-fold cross-validation. We obtained the following equation of the model, of which only bilirubin (TBIL) and creatinine (CREA) were statistically significant

$$\log(\frac{P}{1-P})=1.06+\frac{2.07}{\sqrt{TBIL}}-0.99\cdot ALB-0.018\cdot AGE+5.068\cdot 10^{=4}\cdot HBG^{3}+2.303\cdot \log(CREA)$$

We notice that the risk of microalbuminuria decreased as total bilirubin levels increased (p=0.014).The minus point of this study was the small number of patients, the missing data, and the lack of available information regarding their evolution in time. As a consequence, this study is only a correlational study. These results provide some additional evidence to the well-known hypothesis that high levels of total bilirubin may, indeed, have a protective effect on the development and progression of diabetic nephropathy. 

# Meeting report: P.1.25. MATERNAL BIOCHEMICAL AND HAEMATOLOGICAL PARAMETERS AND NEONATAL OUTCOME

**Authors:** L. Radulescu, O. Munteanu, L. Gaman, V. Atanasiu, M. M. Cirstoiu

**Affiliations:** * “Carol Davila” University of Medicine and Pharmacy, Bucharest, Romania

Mother and child health is an important public health issue that affects the development of family and community as these populations are most exposed to disease and death. Unbalanced nutrition of the pregnant woman is an important external risk factor for both maternal health and that of the newborn.

This study aimed at investigating some parameters of the nutritional status of a group of pregnant women in relation to newborn outcome.

In a retrospective study, 150 pregnant patients and, after birth, their newborns as well, were evaluated. The data analyzed included hematocrit, hemoglobin, erythrocyte count, and glucose levels, total protein, albumin, triglycerides, cholesterol. After birth, the newborns age of gestation, sex, length and birth weight, head circumference, and Apgar score at 1 and 5 minutes were evaluated.

Laboratory tests showed a low maternal mean level of haemoglobin which emphasizes, beside other factors, an unsatisfactory nutritional status and low blood glucose levels. The average newborn indicators were within normal limits. Among the maternal parameters investigated in the mother, hemoglobin correlated positively with the length of the newborn, while the total protein correlated negatively with the weight of the newborn.

The maternal nutritional status plays a crucial role in influencing the growth of the fetus and it is a modifiable risk factor of public health importance in the effort to prevent adverse birth outcomes. 

# Meeting report: P.1.26. THE ASSOCIATION BETWEEN MATERNAL DIETARY MICRONUTRIENT INTAKE AND NEONATAL OUTCOME

**Authors:** L. Radulescu, M. Petran, O. Munteanu, L. Gaman, I. Stoian, V. Atanasiu, M.M. Cirstoiu

**Affiliations:** 1 Carol Davila University of Medicine and Pharmacy, Bucharest, Romania

Pregnancy is associated with an increased demand for nutrients such as iron, copper, zinc, iron, calcium and magnesium and the deficiency of any of these could affect pregnancy, birth and postnatal evolution. 

Evaluation of the nutritional status of the pregnant patient and the postnatal development of the newborn. 

A retrospective study was carried out in which the data of 253 patients and their children were evaluated, by analyzing the values of biochemical parameters in maternal venous blood, umbilical cord blood and capillary blood of the newborn collected at the 5th day of life. The parameters were hemoglobin, hematocrit, iron, copper, zinc, folic acid, iron, calcium and magnesium. 

A significant number of patients had a lower level of Hb, Hct, iron and zinc, while copper and total capacity Iron binding (TIBC) were significantly higher (P <0.001). Newborns had higher levels of iron and zinc and lower levels of copper and TIBC when compared to their mothers, regardless of iron deficiency anemia. In addition, the results suggest micronutrient interactions, which are reflected in the Iron / Zinc, Iron / Copper and Zinc / Copper ratios. 

Considering the relationship of dependence between the newborn’s micronutrient levels and the mother's micronutrient condition, an adequate and balanced nutritional supplementation during pregnancy is necessary for a favorable postnatal development.

# Meeting report: P.1.27. DIFFERENCES BETWEEN THE LEVELS OF MATRIX MATELLOPROTEINASES AND THEIR INHIBITORS IN GLAUCOMA AND DIABETES PATIENTS

**Authors:** I.M. Ciotu, L. Gaman, L. Voinea, V. Atanasiu, A. Constantin

**Affiliations:** 1 Carol Davila University of Medicine and Pharmacy, Bucharest, Romania; 2 Institute of Cellular Biology and Pathology ”Nicolae Simionescu ”, Bucharest, Romania

Glaucoma is an optic neuropathy influenced by many risk factors, like intraocular pressure (IOP), age, gender, the perfusion of the optic nerve and others. The aqueous humor composition and outflow play an important role in influencing the metabolism of adjacent tissues with important consequences in the physiopathology of glaucoma. In aqueous humor matrix metalloproteinases (MMPs) have an important role, permanently remodeling the extracellular matrix of the trabecular mesh, thereby keeping a constant level of IOP. 

The aim of this study is to asses the level of the MMP-2/TIMP-1 and MMP-9/ TIMP-2 complexes in aqueous humor and plasma samples of patients with open angle glaucoma (POAG), with POAG and type 2 diabetes; the control lot consisted of cataract patients. 

Aqueous humor and plasma samples were collected from 50 patients, 15 from POAG subjects, 15 from POAD and diabetes subjects and 20 from cataract subjects. Levels of TIMP -1 and -2 were determined by immunoassay using specific kits. 

We identified MMP-2/TIMP-1 and MMP-9/ TIMP-2 ratio levels in aqueous humor and we compared it with the plasmatic levels of these complexes. We found that the concentration of MMP-2/ TIMP-1 was higher in POAG patients and POAG diabetes patients when comparing with the control lot, but there was no significant difference between the levels of two groups. On the other hand MMP-9/TIMP-2 ratio levels was not statistically significantly raised in neither group. 

Our study suggests that there is an imbalance of the metalloproteinases matrix concerning the pathology of glaucoma, the MMP-2/TIMP-1 having a direct influence in the remodeling process of the trabecular mesh matrix. The role played by other pathologies, like diabetes is not statistically significant.

# Meeting report: P.1.28. THE EFFECTS OF SLEEP DEPRIVATION ON CARDIOVASCULAR PARAMETERS MONITORED BY A NON-INVASIVE METHOD - IMPEDANCE CARDIOGRAPHY

**Authors:** S.M. Lider Burciulescu, A.I. Badarau, M. Buraga, C. Ciornei, R. Papacocea

**Affiliations:** 1 Carol Davila University of Medicine and Pharmacy, Bucharest, Romania

It is known that the total duration of sleep in healthy adults has an average between 7 and 8 hours per night. Sleep deprivation is becoming a problem that affects a big part of the population and involves all social categories. The purpose of our study is to analyze the effect of sleep deprivation on cardiovascular functions on residents and medical students after continuous 24h of sleep loss.

In our study, we have included 20 residents, and medical students-aged between 22-27 years old, 11 men and 9 women. We have evaluated their cardiovascular parameters using impedance cardiography (ICG) and manometric method (for evaluating arterial blood pressure), before and after one night of call duty or after one night of studying in students. 

Surprisingly all subjects have a decrease in their heart rate after one night of sleep deprivation (from mean: 74,6 b/min before to 67,4 b/min after, p = 0,000), also the medium arterial blood pressure is lower after the overnight call (from mean: 93,8 mmHg before to 86,3 mmHg after). Moreover, after one night of sleep loss, cardiac output, stroke volume and cardiac index decreases along with an increase of peripheric vascular resistance and preejection-time. 

Acute sleep loss for 24 hours, due to continuously, intense on-call work or studying, modifies several cardiovascular parameters: heart rate, medium arterial blood pressure, cardiac output, stroke volume cardiac index and vascular resistance.

# Meeting report: P.1.29. EXPANDING THE DETERMINATION OF IODINE FOR OTHER BIOLOGICAL FLUIDS

**Authors:** C. Delia, G. Toma

**Affiliations:** * Alessandrescu-Rusescu National Institute for Mother and Child, Bucharest Romania

 Iodine is an essential trace element of the human body that is indispensable for the synthesis of thyroid hormones that are transported by the bloodstream throughout the body and regulates the processes of growth, brain development, reproduction and metabolism. Although the method was initially described for urine iodine determination, we wanted to see if the use of this method can be extended to iodine from the saliva and blood.

Determination of urinary iodine concentration is based on the "digestion" of the sample with ammonium persulfate followed spectrophotometric measurement of a colorimetric complex at 420 nm after (formed on the basis of the Sandell-Kolthoff reaction.)

Extension for the saliva sample. The same worked 10 times in three different days. The result was more than good, with an average value of 103.5 with a very low coefficient of variation of 0.09%.

Extend the method for the serum sample:

Two ways have been approached: 

• With deproteinization - Was performed with deproteinization with 10% trichloroacetic acid (ATA) - this approach has been abandoned

• Without deproteinization

 This was done in 5 patients, 8 times in 3 different days. The results were also very. The method initially described for determining urine in iodine can be successfully extended.

This study opens new perspectives in assessing iodine status, both at the population level and at the case study level, in individuals with thyroid pathology.

Evaluation and monitoring of diseases due to iodine deficiency in a population can be done in a wider and more complex way. We also consider this test to be useful in breast milk or plasma.

# Meeting report: 2. New Trends in Nutritional Sciences and Food Control KN.2.1. PARADIGM SHIFT IN TASTE SCIENCE

**Authors:** M. Gilca

**Affiliations:** * “Carol Davila” University of Medicine and Pharmacy, Bucharest, Romania

 More and more research studies are revealing that taste, considered until recently to be important only for the detection of nutritious and poisonous substances, have unexpectedly essential roles for health and pathogenesis of various diseases. This paradigm shift in taste science was caused by the recent discovery of extraoral taste receptors in many locations (e.g. stomach, intestines, liver, pancreas, respiratory system, heart, brain, kidney, urinary bladder, adipose tissue, endocrine glands, etc). These taste receptors widely dispersed in the body form a *diffuse chemosensory system*, similar to an iceberg, whose tip is represented by the taste transducers in the oral cavity. Scientists estimate that the functional implications of this discovery may be beyond expectations.

The present work will summarize the extragustative roles of taste, the possible molecular mechanisms involved, and will also offer a new perspective on certain concepts of the ethnomedical epistemology in connection with these scientific facts. The taste of medicinal plants has been used in traditional medicine as criteria for selection of the right remedy, being considered a tool for direct knowledge of the medicinal plant therapeutic potential. How the scientific evidence of extraoral taste receptors may come in support of this traditional medical algorithm will also be discussed.

Integrating the traditional view of taste into the modern science could catalyze a shift in our understanding on the human body interaction with exogenous and endogenous tastants and even reveal new aspects of the therapeutic potential of taste.

# Meeting report: KN.2.2. QUALITY CONTROL TO ASSESS BENEFIT AND SAFETY OF FOOD SUPPLEMENTS

**Authors:** P. Restani, C. Di Lorenzo

**Affiliations:** * Dept. Pharmacological and Biomolecular Sciences, Università degli Studi di Milano, Italy

 The use of food supplements has increased exponentially in the last decades due to the positive feeling of the consumers versus the “natural” products. This phenomenon is particularly evident for products containing botanicals.

The aim of this presentation is an overview of the problems found in quality control, performed for the quantification of molecules responsible for both the possible physiological effects and adverse effects. Different methods have been developed to reach the objectives described above. Some analytical approaches are classified as fast methods and allow a preliminary screening, when the number of samples is high. Other methods are more specific and suitable for reliable quantification: Gas Chromatography and High Performance Liquid Chromatography coupled with different detectors including mass spectrometry. 

Some examples of quality control performed on food supplements containing botanicals will be described during the presentation, citing the developed and validated methods used.

Problems faced in quality control of food supplements will be discussed: among others, the role of matrices in modifying the reliability of methods developed with purified standards. Strategies used to identify illicit additions of ingredients and counterfeits will be also considered.

Quality control is an important tool in protecting the consumers from the consequences of exposure to low-quality food supplements or in the worst cases to active molecules, such as conventional drugs, added to obtain the expected effects (body weight control, energetic effects in athletes, etc.), but which are rarely associated with “natural” ingredients.

# Meeting report: KN.2.3. INVASIVE KNOTWEED SPECIES AS A RICH SOURCE OF ANTIOXIDANTS

**Authors:** L. Pogačnik, N. Poklar Ulrih

**Affiliations:** 1 Biotechnical Faculty, Department of Food Science and Technology, Ljubljana, Slovenia

 Variety of food is important in terms of providing essential nutrients in the human diet. Antioxidants, which allow the organism to defend against the oxidative stress, environmental pollution and other toxic substances, play an important role in the healthy human nutrition. Three alien taxons of knotweed - Japanese (*Fallopia japonica*), Sakhalin (*Fallopia sachalinensis*) and Bohemian (*Fallopia x bohemica*) are abundantly spread all over Europe and represent a big ecological problem, due to their rapid expansion and flexibility, which significantly alter the biodiversity of the area. At the same time, the use of knotweed in traditional Asian medicine is extensive and in many cases successful. Resveratrol is only the most known substance in Japanese knotweed and is already widely used as a dietary supplement, in cosmetics and medicine. Determination of other antioxidants and their bioactivities (e.g. antioxidant, antimicrobial, cytotoxic) from different tissues of knotweed is therefore an interesting topic that cannot change only the attitude of modern society towards these plants, but also contribute to the creation of more effective strategy for limiting their spread. The recent scientific publications are mostly focused on Japanese knotweed, while the research of Sakhalin and the Czech knotweed is limited. Hence, the characterization of these two taxons represent the new challenge due to their altered biological and chemical activity.

*The authors acknowledge the financial support from the Slovenian Research Agency (research core funding No. P4-0121)*.

# Meeting report: KN.2.4. FROM PRO-INFLAMMATORY MOLECULES TO THE BRAIN’S RESTING-STATE CONNECTIVITY. THE FUTURE OF CLINICAL DIAGNOSIS OF DEPRESSION

**Authors:** D. Cirneci

**Affiliations:** * Spiru Haret University Bucharest, Romania

 Although scientists have learned a lot about the brain over the last few decades, the approach to treating mental illness has not kept pace with these findings. Without knowing the causes of the mental illness they were treating, the psychiatrists focused on the subjective symptoms and this approach led them the wrong way. In short, patients have been given names for syndromes or disorders that are not really known to be real entities or to what extent such an entity is different from another. The aim of this paper is to summarize some of the findings from neuroscience which could help finding the mechanisms behind depressive disorders. In the last two decades have been identified biomarkers that can predict response to antidepressant medications, such as hemolytic-encephalopathies barrier function, hormonal dysfunctions, and mechanisms of plasticity. Also, immune analysis allows an early screening of people presenting an increased risk for developing affective disorders, or are in an early stage of these. In addition, imaging and anatomical studies have found alterations in both the structure and function in regions that belong to the some of the brain’s networks, thus suggesting a basis for the cognitive deficits associated with depression. 

# Meeting report: O.2.1. COMBINED EXPERIMENTAL APPROACHES TO ASSESS THE ANTIOXIDANT ACTIVITY IN FOOD

**Authors:** C. Di Lorenzo, M. Badea, F. Colombo, F. Orgiu, A. Miccoli, P. Restani

**Affiliations:** 1 Dept. Pharmacological and Biomolecular Sciences, Università degli Studi di Milano, Italy; 2 Transilvania University of Brasov, Brasov, Romania

Several epidemiological studies have suggested that antioxidant compounds may have a modulating role on risk factors for chronic diseases. These positive effects have been partially associated with antioxidant molecules, including vitamin C and flavonoids, which are present in significant amount in food and beverages (e.g., wine, juices, tea). These compounds can counteract the development of free radicals, involved in several deleterious effects on many biological targets. Considering that the *in vitro* evaluation of antioxidant activity is highly useful to establish a correlation with possible *in vivo* effects, increasing interest has been raised for fast analytical/biological methods. 

The aim of the study was to compare the antioxidant properties of some food and beverage, measured by different analytical approaches. 

Grape juices, wines and food supplements containing acerola and melatonin were firstly tested for their antioxidant activity with a novel method based on an electrochemical biosensor. The method was then compared with spectrophotometric (DPPH and ABTS assays) and chromatographic (High Performance Thin Layer Chromatography) assays. 

Although with some differences, the results obtained with all tests used showed a similar trend: red wines and food supplements containing acerola showed the highest antioxidant activity. An emerging point was the interference of food matrix, which could be responsible for the differences observed intra- and inter-methods.

The use of biosensor integrated with other assays seems to offer a reliable body of data to evaluate the antioxidant activity of food and foodstuffs, reflecting at least partially the *in vivo* protecting potency.

# Meeting report: O.2.2. UNDERSTANDING THE POTENTIAL OF ANTIOXIDANTS: HOW EFFICIENT? WHICH METHODS AND APPLICATIONS?

**Authors:** M. M. Becker, G. Catanante, J-L. Marty, G. S. Nunes

**Affiliations:** 1 BAE Laboratory, Université de Perpignan Via Domitia, Perpignan, France; 2 Program in Biodiversity and Biotechnology, Bionorte, Federal University of Maranhão, Brazil

The antioxidant potential of exogenous natural sources has received great attention due to the increased oxidative stress that has been identified as the cause of the development and progression of various diseases. Similarly, the number of methods and variations for measuring antioxidants in plants has increased considerably. In view of this, the applications and limitations of the main methods, as well as, the potential end- uses of the antioxidants are discussed. Spectrometry, electrochemical and chromatography analytical methods have been used for measuring the antioxidant potential, these assays differ in the mechanism of generation of different radical species and/or target molecules and in the way end products are measured. The uses of antioxidants in pharmacological, medicinal, and therapeutic applications have been intensively reported in the preventing and treatment of several diseases, especially degenerative disorders such as cancer. The use of preservative antioxidants has immense industrial applications such as food additives to increase the oxidative stability, cosmetics to prevent rancidity and aging, rubber and plastic to prevent the degradation and reducing the wastage of raw materials, fuels and lubricants to prevent the oxidation and damage the engines. Due to the antioxidants behaviour that may respond in a different manner to different radical or oxidant sources, the measurement of antioxidants is not a simple process and there is no yet unique simple universal method that can be measured accurately and quantitatively. However, the standardization is longed for unifying quantities and units. Plants with high antioxidant capacity represent an interesting potential for many applications. 

*Scholarship from CAPES/Doctoral Sandwich Program Abroad/Case nº 88881.133624/2016-01*.

# Meeting report: O.2.3. FUNCTIONAL FOOD IN IRRITABLE BOWEL SYNDROM

**Authors:** L. Nedelcu

**Affiliations:** * Transilvania University of Brasov, Brasov, Romania

Irritable bowel syndrome (IBS) is one of the most common functional gastrointestinal diseases that affects in our geographical area round. 14% of the people. Management of this disease is difficult and requires a good doctor-patient communication. The therapeutic decision is difficult and need to consider the intricate etiology of the syndrome. Both the pharmacological and non-pharmacological treatment will be take into discussion. A large number of patients fail to respond to pharmacological therapy. Some diets improve symptoms of IBS, but data supporting their use are limited. Some specific dietary intervention were tested in IBS: dietary fiber supplementation, elimination diets, very low carbohydrate diet, no-gluten diet, Low Fermentable Oligosaccharides, Disaccharides, Monosaccharides, and Polyol Diet (FODMAPs).

There is a growing body of evidence to support the use of a low FODMAP diet in IBS patients. Diet is safe on short-term, but some issues are waiting for the answerer: the type of food, quantity of food, the additive effect of various food, safety and long term efficacy. Low FODMAPs diet reduces IBS symptoms. These carbohydrates are represented by fructose and lactose (apples, pears, watermelon, fruit juices, dried fruit, milk and derivatives), polyols used to produce low calories food, galactan and fructans (wheat, onion, garlic, cabbage, soybeans, broccoli).

The current researches will try to identify fecal bacterial profile of patients who responded to dietary intervention in IBS.

# Meeting report: O.2.4. AMPEROMETRIC BIOSENSOR BASED ON GRAPHENE/FERROCENE CARBOXYLIC ACID/L-AMINO ACID OXIDASE NANOCOMPOSITE FOR THE DETECTION OF L-ALANINE

**Authors:** C. Apetrei, I.M. Apetrei

**Affiliations:** 1 Department of Chemistry, Physics and Environment, European Centre of Excellence for the Environment, Faculty of Sciences and Environment, “Dunarea de Jos” University of Galati, Romania; 2 Department of Pharmaceutical Sciences, Medical and Pharmaceutical Research Centre, Faculty of Medicine and Pharmacy, “Dunarea de Jos” University of Galati, Romania

Detection of optical-active amino acids has received special attention due to its significant impacts on chemical, biological, and pharmaceutical sciences. Among the different analytical techniques, electrochemical biosensors based on enzyme-nanocomposite are attractive due to its simple construction, rapidity, and very good sensitivity. 

In this study, a novel amperometric biosensor for the detection of L-alanine in pharmaceutical samples was developed. The sensing material is based on covalent immobilized L-amino acid oxidase onto ferrocene carboxylic acid functionalized graphene thin film.

L-amino acid oxidase enzyme immobilization was carried out by cross-linking with glutaraldehyde. Ferrocene carboxylic acid was used as redox probe due to its electroactivity. The molecular architecture and interactions among components of sensing layer was determined by using FTIR technique. The morphology of sensing layer was studied by scanning electron microscopy. 

After the biosensor testing towards L-alanine and D-alanine respectively, a larger current response was obtained from L-alanine. The optimizations of supporting electrolyte properties (pH and temperature) and of detection technique parameters (applied potential, stirring rate) were carried out. The biosensor presented an optimal response when a potential of -0.5 V was applied in phosphate buffer solution of pH 8.0. The linear range of the biosensor under the optimum working conditions was from 1.0×10−8 to 1.0×10−4 M with a lower detection limit of 4.2×10−9 M (S/N =3). The interfering effects were studied by standard addition method obtaining an excellent average recovery of 100.5%. 

The biosensor was validated by quantification of L-alanine in pharmaceutical sample, when excellent results were achieved.

*This work was supported by a grant of the Romanian National Authority for Scientific Research and Innovation, CNCS - UEFISCDI, project number PN-II-RU-TE-2014-4-1093*.

# Meeting report: O.2.5. ENZYMATIC BIOFET TRANSISTOR PROCESSING IN A MICROELECTRONIC FOUNDRY

**Authors:** C. Ravariu, F. Babarada, C. Pirvulescu, E. Manea

**Affiliations:** 1 University Politehnica of Bucharest, ETTI, DCAE, Bucharest, Romania; 2 National Institute of Microtechnology, Bucharest, Romania

The Bio-FET transistors are complex electronic devices that integrate in the same chip a Field Effect Transistor (FET) with a sensitive biological receptor. This work starts to present the TCAD techniques used for the simulation and design of the BioFET device, followed by a specialised software ATLAS from Silvaco useful for the physical phenomen an investigation and finishing by few main technological steps that are applied for enzymatic BioFET transistor processing in a microelectronic foundry. By some EDA tools as LEDIT, the layers that will constitute the fabrication masks will be established; then they are transposed on a glass support with chromium metallic traces. The masks and values of the designed parameters have to be correlated with the process fabrication and device size. By some EDA tools as LEDIT, the layers that constitute the fabrication masks that are established; then they are transposed on a glass support with chromium metallic traces. The biodevice description in Atlas program, include an original approach concerning the simulation techniques of the enzymes entrapping onto nanostructured material grown on Silicon wafer.

An enzymatic transistor was implemented in Si, appealing to multiple facilities from a Microelectronics foundry

# Meeting report: P.2.1. DETERMINATION OF BIOACTIVE COMPOUNDS AND ANTIOXIDANT POTENTIAL OF NUTRITIONALLY IMPORTANT AMAZONIAN FRUITS

**Authors:** M M. Becker, V. T. Chagas, C. M. Mandaji, T. A. Malta, L. G. Lima Neto, J-L Marty, T. M. F. F. Mendes, M. Badea, G. S. Nunes

**Affiliations:** 1 Program in Biodiversity and Biotechnology, Bionorte, Federal University of Maranhão, Brazil; 2 Department of Nutrition, Estácio de Sá Faculty, Maranhão, Brazil; 3 BAE Laboratory, Université de Perpignan Via Domitia, France; 4 Transilvania University of Brasov, Brasov, Romania

Findings of the deleterious effects of free radicals on cells and their relation to certain diseases drive the search for foods that can prevent or minimize oxidative damage. Therefore, this work determined the bioactive compounds vitamin C, phenolic compounds and flavonoids, as well as the antioxidant capacity by colorimetric assay for ten Amazonian fruits, popularly known as abiu (*Pouteria caimito*), açaí (*Euterpe oleracea*), bacuri (*Platonia insignis*), biribá (*Rhollinea orthopetala*), buriti (*Mauritia flexuosa*), cupuaçu (*Theobroma grandiflorum*), inajá (*Maximiliana maripa*), monguba (*Pachira aquatica*), pajurá (*Couepia bracteosa*) and uxi (*Saccoglotis uchi*). The vitamin C concentration was determined by redox titration using iodine solution, the total phenolic using a spectrophotometry at 750 nm using standards solutions of gallic acid for calibration curve and the flavonoids content at 425 nm using quercetin in calibration curve. The antioxidant potential was determined by colorimetric assay that is based on the antioxidant activity of the pulp of the fruit diluted in buffer (pH 7.5) that competitively inhibits the superoxide radical O2•- in vitro generated by hypoxanthine/xanthine oxidase system, followed by reduction of the chromosphere nitrobluetetrazolium (NBT) to colored product NBT-formazan (λ=560 nm). The vitamin C levels in the in fruits ranged from 5.20 to 80.0 mg 100 g-1 for the monguba almond and the açaí pulp, respectively. The pulps of biribá and pajurá have been sources and the fruits of açaí, bacuri, buriti, cupuaçu, inajá and uxi foods of high content in vitamin C. The vitamin C content for inajá, monguba and pajurá pulps were presented for the first time in the literature. The concentrations of phenolic compounds obtained for the pulps of the fruits studied reveal a higher content present in the pajurá sample, as well as some agreement between the contents of the other pulps. The pulps of açaí and pajurá presented concentrations of flavonoids of 10.21 and 2.25 EQE 100g-1, respectively. The other fruits showed signs of absorbance that were not quantifiable for the method used. All the fruits studied show antioxidant activity. As expected, the percentage inhibition of the O2•-radical (PIR%) was higher for the acerola pulp (96.39%). However, the pulp of pajurá was shown with a PIR close (95.93%) to this recognized antioxidant source. For the other fruits, PIR values were: buriti (84.28%), açaí (84.21%), cupuaçu (80.45%), abiu (79.33%), bacuri (78.35%), monguba (75.74%), biribá (75.55%) and inajá (73.60%).The uxi pulp showed lower antioxidant action than the other fruits. This study provided new data on the antioxidant activity of native fruits of the Amazon region, as well as new options for assessing antioxidant capacity of complex matrices. Based on this study, it can be said that these fruits are suitable for use in the food and cosmetics industries as well as in pharmaceutical compositions.

# Meeting report: P.2.2. SCREENING OF FUNGI STRAINS PRODUCING TOXINS AND INHIBITION STUDIES OF FUNGAL GROWTH

**Authors:** I. Raut, M. Calin, L. Jecu, M.- L. Arsene, D. Constantinescu-Aruxandei, G. Vasilescu, A.-M. Gurban, M. (Badea) Doni

**Affiliations:** * National Institute for Research & Development in Chemistry and Petrochemistry, Bucharest, Romania

Aflatoxins, ochratoxin A, fumanosin, deoxynivalenol, patulin, zearalenone, trichotheceneas, citrinin and ergotamine represent the most common mycotoxins contaminating the food and the animal feedstuffs, having strongly negative effects over the human/animal health and economy. These mycotoxins are produced by parasitic fungi belonging to *Aspergillus, Penicillium, Fusarium, Claviceps* and *Alternania* genus. Contamination with these toxicants is unpredictable and unavoidable.

Due to their spread in growing crops (corn, wheat and barley), in cereal or animal-based food (meat, milk, eggs, etc) and due to their cytotoxicity, these mycotoxins pose serious problems in controlling and limiting their effects over the human and animal health. 

Taking into consideration all these, several studies have been carried out for the screening of some *Aspergillus, Fusarium, Penicillium* and *Monillia* strains grown in our lab, and for the determination of the mycotoxins levels produced by the selected fungal strains. Levels of aflatoxin B1, ochratoxin, fumanosin and citrinin have been determined by ELISA method.

Bacterial strains able to inhibit the growth of *Aspergillus* fungi strains have been selected and the inhibition level of the selected strains over the fungal growth and mycotoxin production was studied. It has been shown that, by using *Bacillus subtilis* strains it was possible to obtain an inhibition rate of 81% for the production of ochratoxin A, while 74% of inhibition for citrinin production was obtained by using only 25% of bacterial metabolites in the culture medium.

*This work was supported by a grant of the Romanian National Authority for Scientific Research and Innovation, CCCDI – UEFISCDI, project number ERANET-MANUNET II -TOX-HAZ-ASSESS, within PNCDI III and by PN.16.31.01.03, NUCLEU Program*.

# Meeting report: P.2.3. COW MILK ANEMIA – STILL A CHALLENGE IN 21^ST^ CENTURY

**Authors:** E.M. Petran, C. Petran, G. Folea, L.E. Gaman

**Affiliations:** * “Carol Davila” University of Medicine and Pharmacy, Bucharest, Romania

Most frequent form of anemia diagnosed in infants is iron deficiency anemia that can be produced by a various range of causes.

Case management- Male 6 month old infant was sent to the hospital by the general practitioner to investigate the cause of intense pallor and poor weight gain. Medical history reveals that he was fed since birth with whole cow milk and at four month of age apple and cereals were added in his diet. Mother’s medical records show she is beta thalassemic. Laboratory exams at admission in the hospital were: hemoglobin 5g/dL, serum iron 10 μg/dL, TIBC (total iron binding capacity) 512 μg/dL and transferrin saturation 10%. Microscopic exam of the blood showed: microcytosis, hypochromia, target cells and poikilocytosis. Iron deficiency anemia diagnosis was established and oral iron therapy was initiated and proper feeding recommendations were made. The follow up showed a monthly increase of hemoglobin levels until 10 mg/dl, then it stopped. The other laboratory parameters were reevaluated and they revealed hemoglobin 10g/dL, serum iron 85 μg/dL, transferrin saturation 24% and TIBC 220μg/dL and still the presence of target cells and hypochromia. So a hemoglobin electrophoresis was performed and it showed HbA1 90 % and HbA2 5 % and diagnosis of beta thalassemia was established.

Improper feeding from birth of an infant with whole cow milk can impair normal development and weight gain and also can precipitate a severe evolution of an undergoing disease.

# Meeting report: P.2.4. EFFECT OF PHYTOCHEMICAL ANTIOXIDANTS ON HUMAN COLON CANCER CELLS PROLIFERATION AT HYPOXIA AND NORMOXIA

**Authors:** M. Mielczarek-Puta, W. Graboń, A. Barańczyk-Kuźma

**Affiliations:** * Department of Biochemistry, First Faculty of Medicine, Medical University of Warsaw, Poland

Phytochemical antioxidants are used as dietary supplements in cancer prevention but their effect depends on blood concentration. However, the majority of studies performed on cells are conducted with high, non-pharmacological antioxidant concentrations. Besides, cells are usually cultured at non-physiological oxygen (21%), whereas it is known that its level in tumor ranges from 0% to 10%. 

The aim of the study was to evaluate the effect of some phytochemicals at pharmacological concentration (reached in blood after supplementation) on cancer cells viability and proliferation at various oxygen levels. Human primary (SW480) and metastatic (SW620) colon cancer cells were cultured at hypoxia (1% oxygen) and physiological (10% oxygen) normoxia without (control) and with single compound: quercetin, epigallocatechin gallate, lipoic acid, hydroxycitric acid, and their mixture. The cell viability and proliferation were determined by trypan blue dye exclusion and MTT assays, respectively. 

The viability of each line ranged from 80% to 97%, and it was independent on the compound and oxygen level. At hypoxia the cell count was similar for both lines but lower than for the controls, whereas at normoxia it was decreased only for metastatic cells. The lowest cell count was observed with the mixture of compounds, at both oxygen levels. 

Our results indicated, that the studied compounds were not cytotoxic at pharmacological levels but their cytostatic effect depended on cell line type and oxygen availability.

# Meeting report: P.2.5. IN VITRO CHARACTERIZATION OF BIOACTIVITIES OF FLOWER AND RHIZOME EXTRACTS OF BOHEMIAN AND JAPANESE KNOTWEED

**Authors:** T. Bergant, N. Poklar Ulrih, J. Viktorová, T. Rum, L. Pogačnik

**Affiliations:** 1 Biotechnical Faculty, Department of Food Science and Technology, Ljubljana, Slovenia; 2 University of Chemistry and Technology Prague, Department of Biochemistry and Microbiology, Czech Republic

The aim of our research was to compare the antimicrobial, antidiabetic, anticancer and antioxidative activities of Japanese (*Fallopia japonica*) and Bohemian (*Fallopia x bohemica*) knotweed flower and rhizome ethanol extracts. Lyophilised extracts were dissolved in methanol. Regarding antimicrobial activity, the samples were not able to inhibit bacterial growth, however they had an effect on the yeast growth, rhizome extracts being more effective than the flower ones. The extracts were able to inhibit α-amylase in an antidiabetic assay as well, the rhizomes were more effective than the flowers. Since the knotweed extracts are already used in pharmaceutic industry, the possibility of toxicity is very low. Their hemolitic assay proved safety of our samples towards normal blood cells; moreover, we determined cytotoxicity to tissue cell lines based on resazurin assay. We used three cancer cell lines (HepG2, HeLa, PaTu) and one non cancer cell line Hek. We found that PaTu cell line was the least sensitive towards all knotweed extracts, whereas Hek cell line was the most sensitive. Flower extracts of both taxons were less toxic towards cells than the rhizomes ones. Finally, the highest antioxidative activities, based on cellular antioxidative activity assay with HepG2 cell line, were determined in flowers of both taxons. We believe that the flowers and rhizomes of knotweeds indicate new source of antioxidants for prevention of diabetes and cancer diseases.

# Meeting report: P.2.6. IDENTIFICATION AND QUANTIFICATION OF ANTIOXIDANTS IN INVASIVE KNOTWEED SPECIES

**Authors:** M. Korenčan, M. Skrt, N. Poklar Ulrih, L. Pogačnik

**Affiliations:** 1 Biotechnical Faculty, Department of Food Science and Technology, Ljubljana, Slovenia; 2 Centre of Excellence for Integrated Approaches in Chemistry and Biology of Proteins, Ljubljana, Slovenia

The differences of antioxidant content and their activities in Japanese knotweed (*Fallopia japonica*), Sakhalin knotweed (*F. sachalinensis*) and their hybrid Czech knotweed (*F. x bohemica*) were examined in this research. We prepared ethanol extracts of different tissues (rhizomes, leaves, stems and flowers) of all three taxons. The highest antioxidant capacity (AOC), determined by ABTS assay, was found in rhizome sheaths of Japanese knotweed (3.0 mmol TEAC/g), while the lowest AOC was detected in stems of the same taxon (0.23 mmol TEAC/g). The highest prooxidant activities, determined by reduction of Fe3+, were detected in rhizome sheaths and flowers of all taxons (1 – 2 mmol/g expressed as ascorbic acid equivalent). The presence of resveratrol, polydatin, catechin and epicatechin was determined by a HPLC/DAD system. As expected, Japanese knotweed rhizome is the richest source of resveratrol (250 µg/g) and polydatin (23.5 mg/g). However, the highest content of catechin (13.3 mg/g) was determined in rhizomes of Sakhalin knotweed, while the richest source of epicatechin is flowers of Czech knotweed (27.1 mg/g). Finally, we examined the stability of resveratrol and polydatin in different temperature conditions. It turned out, that both compounds are stable up to 48 hours at temperatures up to 60 °C. We believe that the results of this research can help to use invasive knotweed species as new dietary supplements.

*The authors acknowledge the financial support from the Slovenian Research Agency (research core funding No. P4-0121)*.

# Meeting report: P.2.7. RECOVERY OF SILYMARIN FROM THE BYPRODUCT RESULTED FROM COLD-PRESSING OF MILK THISTLE SEEDS

**Authors:** C. V. Popa, S.C. Litescu, A.F. Danet, G. Badea, A. E. Bratu

**Affiliations:** 1 University of Bucharest, Department of Organic Chemistry, Biochemistry and Catalysis; Romania; 2 Centre of Bioanalysis, National Institute for Biological Sciences, Bucharest; Romania; 3 University of Bucharest, Department of Analytical Chemistry

Milk thistle (*Silybum marianum* (L.) Gaertner) is native to the Mediterranean area and recommended in traditional European and Assian medicine, mainly for treatment of liver disorders. Active component of this plant is silymarin which is a mixture of flavonoids including taxifolin, silychristin, silydianin, silybinin A, silybinin B, isosilybinin A and isosilybinin B. From Milk thistle seeds it is obtained valuable oil by cold-pressing and result a byproduct reach in silymarin (about 1.5 - 4.0 %). The aim of this work was to develop a method for recovery of silymarin from byproduct using different solvents (ethanol, methanol, acetone, ethyl acetate, water and mixture of them). It were studied the influence of the temperature, of the time, and of the ultrasounds and microwaves on the extraction. The yield of silymarin extraction from byproduct was evaluated by HPLC - DAAD and HPLC - MS methods. The total phenols and antioxidant capacity of the extracts were determined.

*Financial support from the UEFISCDI, PN-III-P2-2.1.-BG 2016-0119, project no 13BG/2016 is gratefully acknowledged*.

# Meeting report: P.2.8. ANTIOXIDANT CAPACITY AND TOTAL PHENOLS CONTENT CHANGES OF BROCCOLI SPROUTS AFTER EXOGENOUS SUPPLY WITH NANO SELENIUM

**Authors:** M. Tocai, S. Cavalu, V. Laslo, P. Burescu, S.I. Vicas

**Affiliations:** 1 University of Oradea, Biology Doctoral School, Romania;; 2 University of Oradea, Faculty of Environmental Protection,Oradea, Romania; 3 University of Oradea, Faculty of Medicine and Pharmacy, Oradea, Romania

The relation between the minerals content of plants and human health benefits was proven by previous data from literature. Selenium is an essential trace element for human health due to its different biological activities. 

Our aim was to investigate the effects of nano-selenium (NSe) supply on broccoli sprouts from point of view of growth parameters, total phenols content and antioxidant capacity of selenium-enriched broccoli sprouts.

The NSe particles were produced by chemical reduction of NaHSeO3 with glucose. Physico-chemical characterisation of NSe was performed by UV-Vis spectroscopy and Dynamic Light Scattering. Broccoli seeds were germinated in the plastic boxes, sprinkled every day with different concentrations of nano-selenium solution (10, 50 and 100 mg/L) for 9 days. The length of shoots and roots were measured, and the total phenols content was determined by Folin-Ciocalteu method, while antioxidant capacity was evaluated by DPPH assay.

By NSe supply, the growth parameters of broccoli sprouts were not affected compared with the control sample. Total phenol content of shoots was not affected by treatment with NSe, but 50 and 100 mg/L NSe supply increased the total phenols content of roots compared with the control. The antioxidant capacity of shoots was increased significantly (P=0.05) in all the samples treated with NSe. With respect to the roots, only the treatment with 10 mg/L significantly increased the antioxidant capacity.

Using NSe particles as fertilizer, selenium-enriched broccoli sprouts can be obtained, with positive effects on human health. 

*This work was supported by a grant of the Romanian National Authority for Scientific Research and Innovation, CNCS/CCCDI - UEFISCDI, project number PN-III-P2-2.1-PED-2016-1846, within PNCDI III*.

# Meeting report: P.2.9. EXPERIMENTAL STUDIES USING ELECTROCHEMICAL DETECTION OF IODIDE COMPOUNDS

**Authors:** L. Chelmea, C. Adochite, L. Floroian, M. Badea

**Affiliations:** 1 Faculty of Medicine; Transilvania University Brasov, Romania; 2 Faculty of Electrical Engineering and Computer Sciences, Transilvania University Brasov, Romania

Iodine is a micronutrient of high importance for the health and good development of individuals. It is contained in many foods, but it is also part of the chemical component of oceans and soil. Iodine deficiency is still a problem of humanity, which has a serious repercussion on our health. It is therefore important to have specific, fast and cheap methods of detection from different samples.

This work aims to identify optimal parameters for potassium iodide (KI) detection from different medium, to be applied to real samples: water, biological fluids.

Results showed a significant difference in electrochemical results, depending on pH values of the mixture and also the time which influences the compounds stability. Differential pulse voltammetry using carbon printed sensors is an important analytical tool which has a wide range of applications in the food, medicine, toxicology and other domains.

# Meeting report: P.2.10. THE OCULAR PROTECTIVE EFFECTS OF PLGA LUTEIN NANOPARTICLES IN THE OBESE WISTAR RATS TREATED WITH SYSTEMIC GLUCOCORTICOIDS

**Authors:** A. Dinu, M. Balaban, B. Virgolici, B. Calenic, R. Barac, I. Ionascu, D.A. C. Stefan, M. Daniela, A. Totan, M. Mohora

**Affiliations:** 1 "Carol Davila" University of Medicine and Pharmacy Bucharest; Romania; 2 University of Agricultural Sciences and Veterinary Medicine, Bucharest,Romania

Lutein is an antioxidant found in ocular layers. Glucocorticoids cause cataract. Poly(lactic-co-glycolic acid), PLGA are biodegradable polymers used as drug delivery system.

The purpose of the study was to evaluate the ocular protective effects of PLGA lutein nanoparticles in rats treated with systemic glucocorticoids.

The study was conducted on 25 males, 12 months aged Wistar rats divided in groups of 5 rats each: the control group (C) with standard diet and no treatment and groups with prednison treatment (1 mg / kg body weight), with and without PLGA nanoparticles loaded with lutein (1 mg / kg body weight), fed either with standard or fat diet. The duration of treatment was 6 weeks and it was done by gavaje. The eye examination was done by two ophtalmologists as a blind test and photos of the eyes were made.

The rats treated with prednison developed cataract and uveitis and the most severe injured eye was observed in the group with high caloric fat diet and no lutein as a supplement. In all rats, the co-administration of lutein as PLGA nanoparticles with prednison prevented the lens, retina and choroidal injuries.

The PLGA lutein nanoparticles assured global eye protection, reducing the ocular side effects of prednison.

# Meeting report: P.2.11. DESIGNING OF A FLOW-ANALYTICAL SYSTEM FOR TOXINS DETECTION

**Authors:** A.-M. Gurban, D. Constantinescu-Aruxandei, M. Călin, I. Răut, G. Vasilescu, M.-L. Arsene, L. Jecu, F. Oancea, M. (Badea) Doni

**Affiliations:** * National Institute for Research & Development in Chemistry and Petrochemistry, Biotechnology Department, Bucharest, Romania

Diary milk is prone to contamination with toxins from microorganisms, the most dangerous ones being the Shiga toxin produced by *Escherichia coli* and mycotoxins, especially aflatoxin M1 (AFM1). Also, the Shiga toxin is the major cause for the haemolytic-uremic syndrome (HUS) and the mycotoxins cause hepatotoxic and carcinogenic effects. It is therefore important to develop rapid, selective, sensitive and cost-effective assays for measuring the level of toxins and tracing the source of contamination. 

Up to now different methods have been reported for the detection of AFM1, such as thin-layer chromatography (TLC), high-performance liquid chromatography or enzyme-linked immunosorbent assays (ELISA). 

A flow injection immunoassay system has been designed for the electrochemical detection of AFM1 from milk. The AFM1 was determined by an indirect competitive assay, the mixture consisting of antigen, tracer and antibody being incubated off-line until equilibrium was reached. This mixture was introduced into a flow-injection system where the complex antigen-antibody is trapped onto a Protein G column and the tracer was eluted. AFM1 labelled with peroxidase was used as tracer and the peroxidase activity was measured using 3,3`,5,5`-tetramethylbenzidine as substrate. A screen printed carbon electrode (DropSens) inserted in an appropriate flow cell was used for detection.

Further, an on-line analytical system will be designed for assessment of AFM1 in milk and dairy products based on a combined SPR screen-printed sensor. It is expected to obtain more precise and reliable detection of mycotoxin in milk using both SPR and electrochemical detection.

*This work was supported by a grant of the Romanian National Authority for Scientific Research and Innovation, CCCDI – UEFISCDI, project number ERANET-MANUNET II -TOX-HAZ-ASSESS, within PNCDI III and by PN.16.31.01.03, NUCLEU Program*.

# Meeting report: P.2.12. RECYCLING OF WASTE FROM AGRI-FOOD INDUSTRIES: THE REDVALUE PROJECT

**Authors:** M. Pérez Fernández, P. Pires, M. Vaz Vehlo, V. Andreu, O. Martín Belloso, M. J. Rodríguez Yoldi, J. A. Couto Teixeira, M. A. Cubero Márquez, G. Catanante, J-L. Marty

**Affiliations:** 1 INTERQUIMICA, Logroño, Spain; 2 Instituto Politecnico de Viana do Castelo, Portugal; 3 AKINAO, Perpignan Cedex, France; 4 Universitat de Lleida, Departamento de Tecnología de de Alimentos, Lleida, Spain; 5 Universidad de Zaragoza, Fac. Veterinaria. Unidad de Fisiología, Zaragoza, Spain; 6 Universidade do Minho, Departamento de Engenharia Biológica, Braga Portugal; 7 INDULLEIDA Lleida, Spain; 8 Université de Perpignan Via Domitia, Perpignan Cedex, France

The process of processing raw materials in the agro-food industry generates a large amount of waste in various forms: bark, skins, stems, seeds, and pulps among others. This waste can make it possible to obtain compounds that are beneficial to health and of high added value with a safe potential market. These secondary products derived from a manufacturing process are called «food-by-products». Multiple studies have been published on the antimicrobial, oxidants and anticancer properties of extracts from forestry and food products.

The specific objective of the project is to evaluate the potential of selected extracted from food-by products. Depending on their chemical characteristics, their activity will be tested as anticancer potentials, antioxidants, antimicrobials, and dietetics or as natural pesticides in organic farming.

The main objective is to find out new active substances, which are a specific activity. In fact, the current environmental and health problems which are cancer, cell oxidation, obesity and accumulation of pesticides in the crop are great concerns to all the SUDOE territory. The aim is to obtain a classification of the substances extracted according to their activity and a complete validation of the methodology used

REVALUE is a project co-financed by the European Union through the Interreg IV B SUDOE (South-West Europe) Territorial Cooperation Programme. It is integrated into the first priority of the Programme: “promoting research, technological development, and innovation”. The presented opinions only compromise the partners and, consequently, in no way represent the opinion of the Territorial Cooperation Programme’s management bodies.

# Meeting report: P.2.13. ANTIOXIDANT CAPACITY EVALUATION OF SOME FRUIT EXTRACTS BY USIG A NANO OXIDES BASED METHOD

**Authors:** C. V. Popa, A.F. Danet, S.C. Litescu, A. P. Visan

**Affiliations:** 1 University of Bucharest, Department of Organic Chemistry, Biochemistry and Catalysis, Romania; 2 University of Bucharest, Department of Analytical Chemistry, Romania; 3 Centre of Bioanalysis, National Institute for Biological Sciences, Bucharest, Romania

It was studied an innovative oxide nanoparticle-based method for rapid detection of antioxidants in food and other biological samples. The method is based on the use of immobilized oxide nanoparticle on filter paper. The oxide nanoparticle changes colour after interaction with antioxidants by means of redox and surface chemistry reactions. The sensors are scanned by a conventional office scanner and the red, green, blue (RGB) colour breakdown is analyzed. By plotting the inverse of blue colour intensity as a function of log of a standard antioxidant concentration it is obtained a calibration curve. Several antioxidants have been used as standards: caffeic, chlorogenic, gallic, ellagic, rosmarinic acids, quercetin and rutin. The calibration graphs are linear in the domain 5 x 10-4 – 6 x 10-3 M. The best results were obtained with MgO, ZnO and CeO2 nanoparticles. 

The developed assay is very fast, it could be easily automated, and the necessary instrumentation is inexpensive. 

*Financial support from the UEFISCDI, for project PN-II-Partnerships no 145/2014 is gratefully acknowledged*.

# Meeting report: P.2.14. DEVELOPMENT OF PEG-APTAMER ASSISTED IMPEDIMETRIC APTASENSOR FOR ZEARALENONE DETECTION

**Authors:** V. Sunil Kumar, K. Yugender Goud, G. Catanante, K. V. Gobi, J- L Marty

**Affiliations:** 1 BAE Laboratory, Université de Perpignan Via Domitia, Perpignan, France; 2 Department of Chemistry, National Institute of Technology, Warangal, Telangana, India

We have developed and designed a disposable, cost effective, label-free electrochemical aptasensor for zearalenone (ZEN). At first, we washed the screen printed electrode (SPCE) surface by sulphuric acid solution followed by electrochemical deposition of carboxy-amine functionalized Poly (ethylene glycol) 2-aminoethyl ether acetic acid (NH2-PEG-COOH) on to the surface of the SPCE. Afterwards, aptamers were immobilized onto the electrode surface by using EDC-NHS covalent binding chemistry. The aptasensor fabrication steps were characterized by cyclic voltammetry (CV) and electrochemical impedance spectroscopy (EIS). The important parameters like aptamer concentration incubation time, PEG concentration were optimised by using EIS technique. 

Electrochemical impedance experiments were performed by using the optimized parameters with varying concentrations of analyte. For this purpose, ZEN was dispensed on the aptamer bound SPCE electrodes (SPCE/PEG/Apt) at various concentrations (0.3125, 0.625, 0.125, 2.5, 5.0, 10 and 20.0 ng mL−1) with an incubation time of 3 h. After the incubation, the electrochemical impedance spectra were recorded and the results obtained with the aptamer based on SPCE/PEG/Apt electrode. The corresponding Rct values were calculated and presented as Δ ratio against ZEN. The analytical performances of the developed aptasensors were tested by taking ZEN as model analytical molecule. Good limit of detection (LOD) value was obtained (0.3 ng mL-1), in the dynamic concentration range from 0.3125 to 20 ng mL-1 for ZEN detection. The applicability of the designed aptasensors was demonstrated for ZEN detection in corn samples. Good recovery values were obtained.

*This work was supported by ARPE 2014-2017 project: COCOTA 2014-002784/01/02. Authors would like to thanks EUPHRATES Program for ERASMUS Mundus Doctoral Fellowship*.

# Meeting report: P.2.15. COMPARATIVE STUDY ON THE EFFECTS OF WHEAT FLOUR SUBSTITUTION WITH GRAPE SEED POWDER (GSP) AND GRAPE POMACE POWDER (GPP) ON THE RHEOLOGICAL PROPERTIES OF BREAD DOUGH

**Authors:** O.B. Oprea, L. Apostol, L. Gaceu

**Affiliations:** 1 Transilvania University of Braşov, Braşov, Romania; 2 National Institute of Research and Development for Food Bioresources, Romania

Wine production is one of the most important agricultural activities throughout the world. The wine-making process involves the generation of a significant amount of waste and wastewater. These residues should be addressed for recycling or treatment before being returned to environment. Winemaking generates different residues characterized by high contents of biodegradable compounds and suspended solids. Grape marc and seeds can be used to obtain anthocyanin colorants or oils, and catechin polymers, respectively. Antioxidant activity of polyfenols was reported. 

Grape seed powder (GSP) and grape pomace powder (GPP) were added at 0%, 5%, 10%, 15% flour substitution level for different bakery recipes. Rheological parameters of the dough were analyzed by using Mixolab equipment, using Chopin, at tank temperature 30 oC, mixing speed 80 rpm, dough weight: 75 g. 

Typical records were: water absorption, dough weight, tank temperature, mixing speed, torque, amplitude, dough stability. The comparative study shows a good behaviour of the dough obtained by addition of GSP and GPP, opening new possibility to prepare new functional food products in the area of bakery industry.

The final products after baking were analyzed taking in consideration: final specific volume, shape coefficient, porosity, elasticity and brittleness of the core products. The final specific volume, shape coefficient and porosity were determined by using Image Pro software application. Wheat flour substitution with GSP and GPP decrease rheological properties of the bread dough, so maximum GSP addition was fixed at the level of 5% and GPP addition was fixed at the level of 10%.

# Meeting report: P.2.16. CHARACTERIZATION OF ANTIOXIDANT ACTIVITY OF PLANT EXTRACTS USING SPECTROPHOMETRY AND BIOSENSORS

**Authors:** G. Paraschiv, O. B. Oprea, L. Gaceu, L. Floroian, Gh. Coman, M. Badea

**Affiliations:** * Transilvania University of Braşov, Braşov, Romania

It is well known that plants have a considerable amount of antioxidants. The technology used for obtaining plant extracts allows a good preservation of antioxidant components. 

This paper was focused on determination of antioxidant activity of *Pleurotus Ostreatus*. Pleurotus was procured from a specific farm from Harman, Brasov County. The material was dried in a convective drier and the humidity content was reduced from 56% to 3%. Next, the alcoholic extract was obtained by using ethanol 96% (sample: alcohol= 1:10/ w:v) in Timatic extractor, built by Tecnolab srl., Spelo, Italy. Extraction method is based on cyclic percolation at 8 bar pressure.

For the characterization of antioxidant activity, two methods were tested: spectrophotometry - that characterizes the absorption of light at different wavelengths and biosensor detection of antioxidant activity - using electrochemistry. Analyses were done at pH 7. In agreement with the antioxidant definition, the signal corresponds to the ability of the sample to donate electrons. Three level of concentration were tested using both methods. 

The results show similar qualitative results and open new opportunities for valorising the antioxidant potential of *Pleurotus Ostreatus* extracts. Future researches will be done with different other extracts made from *Pleurotus* wastes resulted in processing industries.

# Meeting report: P.2.17. THE STUDY OF THE BIOLOGICAL EFFECTS OF SOUND (MUSIC) ON THE CELLS CULTURE

**Authors:** M. Badea, L. Floroian, D. Ursutiu, C. Samoila, S. Dragulin

**Affiliations:** 1 Faculty of Medicine, Transilvania University of Braşov, Braşov, Romania; 2 Faculty of Electrical Engineering and Computer Science, Transilvania University of Brasov, Romania; 3 Faculty of Material Science and Engineering, Transilvania University of Brasov, Romania; 4 Faculty of Music, Transilvania University of Brasov, Romania

Music therapy has been shown to improve anxiety in a variety of acute medical settings. Human beings, in the habitat environment, are not subjected to pure frequencies. In fact we are exposed at a sum of noise, sounds and music without knowing totally their effect on our cells.

The study of the biological effects of sound (music) on the cells culture there is a strong trans-disciplinary and inter-disciplinary research field. 

There are scientific studies to evaluate different in situ antioxidant processes that may play a role in the toughening phenomenon initiated by low level noise exposure. There were studied different enzyme activities in stria vascularis and organ of Corti fractions from cochleae of chinchillas exposed to a sound conditioning paradigm.

The studies of ultrasound (high frequency sound) confirmed that sound energy could determine specific changes of some enzyme activities and cell metabolism.

Further experimental studies need to be performed to underline and demonstrate the effect of different sound frequencies in cell cultures.

# Meeting report: P.2.18. MEDITERRANEAN DIET AND HEALTHY LIFESTYLE

**Authors:** M. Badea, L. Gaman, L. Floroian, Lluis Serra Majem

**Affiliations:** 1 Transilvania University of Braşov, Braşov, Romania; 2 “Carol Davila “University of Medicine and Pharmacy, Bucharest, Romania; 3 Faculty of Electrical Engineering and Computer Science, Transilvania University of Brasov, Romania; 4 Instituto Universitario de Investigaciones Biomédicas y Sanitarias de Las Palmas, Universitad Las Palmas de Gran Canaria, Spain

Foods recommended in the Mediterranean diet are healthy and beneficial to reducing the risk of disease.

Scientific studies demonstrated the beneficial and preventive role of Mediterranean diet in the occurrence of several diseases as cardiovascular diseases, chronic neurodegenerative diseases and neoplasms, diabetes and obesity.

The work aims to underline the characteristics of Mediterranean diet and the advantages for different human categories.

Based on scientific evidence, Mediterranean diet combined with a healthy lifestyle (sport, healthy living habits) indicated lower risk of depression, extending longevity, increase the quality of life.

During the last years, it was identified the increasing activities in order to revitalize the Mediterranean diet by improving the current perception of people not only as a healthy diet but also a sustainable lifestyle model, with particularities and variations due to the several specific traditions of each country.

# Meeting report: P.2.19. STIMULANTS FOR LEARNING: THEIR USE AMONG YOUNG PEOPLE AND HOW THEY ARE REFLECTED IN ROMANIAN MEDIA

**Authors:** L. Mesesan, L. Rogozea, M. Badea

**Affiliations:** * Transilvania University of Braşov, Braşov, Romania

Cognitive stimulants are nowadays an increasingly popular topic for young people in Romania, but especially for students from abroad. Although used to treat neurological or psychiatric disorders, a large number of patients with no health problems use these pills to increase their cognitive ability, making them more alert, more focused, smarter. In the learning process, these psychostimulants could be the quick way to assimilate new information by enhancing brain metabolism and protecting it from the harmful action of free radicals.

The main objectives of this work were to determine the frequency of nootropic use by providing questionnaires to high school students and students at the Faculty of Medicine and the Faculty of Food and Tourism (Transilvania University of Braşov) and to analyse the level of knowledge concerning the effects of nootropics through Romanian media analysis.

Preference for study participants indicates affinity for nutraceuticals in 84 cases for use in students and 125 for students, who chose these incentives to increase learning attention, increase school results and calm before exams.

In the case of 19% students and 26% students there were self-reported adverse effects on the use of psychostimulants. Among these, the most common were: nervousness, insomnia and headaches.

With regard to informing young people about the adverse effects of cognitive stimulants, 33% of the articles dealing with this issue were noted, which implies a need for campaigns to inform pupils and students about how psychostimulants can help them, but especially with regard to the adverse effects and contraindications they have, based on the scientific studies. In this regard, a first step is to provide information seminars in schools, with informative leaflets and debates with the students on this topic.

# Meeting report: P.2.20. COMPARATIVE ANALYSIS OF THE ANTIMICROBIAL ACTIVITY OF EXTRACTS FROM "BRASSICA HIRTA", "URTICA DIOICA", "VITIS SEMEN", "BRASSICA NAPUS OLEIFERA" AND "GLYCINE MAX". STUDY CASE ASPERGILLUS BRASILIENSIS.

**Authors:** B. Oprea

**Affiliations:** * Faculty of Food and Tourism, Transilvania University of Braşov, Braşov, Romania

Extending the shelf life of the bakery products is one of the most important priority in bread making research area. Antimicrobial activity of different vegetal extracts upon some fungi can be used in order to minimise activity of some fungi which depreciate bakery products.

The paper shows a comparative analysis of the extracts from "*Brassica hirta", "Urtica dioica", "Vitis semen", "Brassica napus oleifera"* and *"Glycine max"* from the point of view inhibition of *Aspergillus Brasiliensis*. The extracts were obtained by using a rapid extractor Micro Timatic, Tecnolab, Italy. The working cycle is fully automatic and alternates a dynamic phase, obtained in programmed pressure at aprox. 8 bar, and a static phase necessary for the transfer of the substance in the solvent extractable. 

During the stance phase generates a forced percolation which, thanks to the programmable recirculation ensures a continuous flow of solvent to 'interior of the plant matrix thus avoiding over-saturation and the formation of preferential channels, thereby ensuring a total extraction of the active of the product itself. The solvents used were: alcohol, water, glycol, and temperature of processing was aprox 20 degree.

The method used for antimicrobial activity evaluation was agar diffusion method, using 90 mm diameter aseptic Petri dishes and 8 mm filter paper discs. The discs were impregnated with extracts, in different quantities.

The results show a big potential in the use of extracts of „*Brassica napus oleifera*” for extending the shelflife of the bakery products. 

# Meeting report: P.21. DEVELOPMENT OF AMPEROMETRIC ACHE-BASED BIOSENSORS TO DETECT MICROCYSTINS IN AQUATIC ENVIRONMENTS

**Authors:** L. A.S Souto, F..G.S. Silva, J-L Marty, M. Badea, G. S. Nunes

**Affiliations:** 1 Group of Studies and Environmental Analysis, Federal University of Maranhão Federal University of Maranhão, Brazil; 2 BAE Laboratory, Université de Perpignan Via Domitia (UPVD), Perpignan CEDEX, France; 3 Faculty of Medicine, Transilvania University of Brasov, Romania

Microcystins (MC-LR) make part of a class of hepatotoxins produced by cyanobacteria in surface water. Some studies have shown that MC-LR inhibits the action of intracellular proteins, alkaline phosphatases, but it has also been proved that such pollutants also increase the acethylcholinesterase (AChE). In this study amperometric biosensors were developed, electrochemically characterized and applied for indirect MC-LR detection in water samples. For the biosensor preparation, a graphite powder paste containing hidroxicetilcelulose (HEC), bovine serum albumin (BSA) and glutaraldehyde (Glu) was prepared. AChE enzymes extracted from bovine erythrocyte (EB), electric eel (EE) and original or genetically modified *Drosophila melanogaster* were tested as biorecognition elements. Also, butyrylcholinesterase (BChE) obtained from human serum was tested. A portion of the sensitive paste was deposited in the graphite working electrode and electrochemical assays involving differential pulse voltammetry (DPV) and cyclic voltammetry (CV) were performed in order to characterize the obtained biosensors. Some operating conditions, such as working potential, electrochemical mediator, pH and substrate concentration were optimized. Enzyme activation tests were performed through chronoamperometric measurements and relative enzyme activation (% RA) was calculated as a function of MC-LR content. The results have shown that EE-AChE has presented relatively high % RA (> 10%) in presence of traces of MC-LR. The biosensor can operate in a large working range (0.5 to 100 μg.L-1).

(EE)AChE-based biosensor has provided a precise (CV ~ 8.32%) and sensitive (LOD of 0.27 μg.L-1 and LOQ 0.91 μg.L-1) analytical system for the MC-LR detection in aquatic environments. 

Such analytical device was then used for the field monitoring of this pollutant in seven sampling points of Bacanga River, an important aquatic ecosystem of São Luis, Maranhão State, Brazil. The results indicated that there was no significant contamination in all points investigated. The biosensor proved to be a powerful tool for alarm in relation to aquatic contamination.

# Meeting report: P.2.22. BORDER ZONES INSIDE THE ARTERIAL VASCULARISATION OF THE CEREBRAL HEMISPHERES

**Authors:** B.M. Cristea, L. Stroica, M. Radoi

**Affiliations:** * “Carol Davila” University of Medicine and Pharmacy, Bucharest, Romania

Border zones, situated at the boundary between the vascularisation territories of two or more large cerebral arteries, are sensitive to modifications in hemodinamic conditions and often constitute the location of vascular ischemic lesions. Strokes located at border zone levels can associate clinical manifestations characteristic to the injury of two large cerebral arteries, or on the contrary, symptoms can present as mild.

The study was set out to monitor two main directions: the anatomical study and the imaging study. Taking into consideration the distribution of cerebral arteries at a superficial level, the present anatomical study was able to facilitate the organisation of border zones, as follows: on the superolateral side, on the medial side and on the inferior side.

Due to the study performed by injecting coloured substances, we have established the fact that at central level these border zones are located inside white matter – especially inside the internal capsule – in the caudate and lenticular nuclei.

Imaging investigation methods – computed tomography and magnetic resonance imaging– are at present the only ones able to provide information not only of a morphological nature but also of a functional one regarding the arterial vascularisation of the brain. At cortical level, the parieto-occipital region is the most affected, because of its location at the limit between the territories of the three sources of cerebral vascularisation.

# Meeting report: P.2.23. BIOCHEMICAL APPROACH OF THE MILD TRAUMATIC BRAIN INJURIES

**Authors:** T. Papacocea, I. A. Bădărău, M. M. Buraga, I. R. Papacocea

**Affiliations:** 1 “Carol Davila" University of Medicine and Pharmacy, Neurosurgery Department, “St.Pantelimon” Emergency Hospital Bucharest; 2 “Carol Davila" University of Medicine and Pharmacy Bucharest, Physiology Department I; 3 “Carol Davila" University of Medicine and Pharmacy Bucharest, Physiology Department II

Traumatic brain injuries (TBI) increased in last decades both in Romania and worldwide; However, their consequences consisting of trend and long term disabilities create important economic and social costs. An early evaluation of short term prognosis can be indicated by several biomarkers, but such a biomarker or a cluster of biomarkers were not clearly described. This is why we are trying to use an association of blood parameters to evaluate short term prognosis og the TBI.

A number of 45 patients with mild TBI according to Glasgow Coma Scale (GCS) at admission were included in our study. Control group consisted of 12 cases of minor orthopedic complains. The brain injury was documented by CT or IRM and none of these need neurosurgical intervention. Classical biochemical parameters included plasma glucose, urea, creatinin, electrolytes and these were determined using a Pathfast Compact immune Analyzer ver. 2.00 Mitsubishi.To compare study group to control was determined mean ± SD; the differences between groups were analyzed using t-tests and correlation analyze was considered significant when p<0,05. Results showed significantly reduced values (p<0,05) for plasma Na and glucose versus control. The relationship of these changes to early evolution was determined.

Even though plasma Na and glucose decrease seem to predict a poor evolution in patients with mild TBI, given to the small number of cases these determinations should be confirmed by further studies.

# Meeting report: P.2.24. INFLUENCE OF THE TECHNIQUE AND EXTRACTION SYSTEM ON THE HYDROALCOHOLIC EXTRACTS CHARACTERISTICS OF *EUGENIA CARYOPHYLLATA*

**Authors:** D. Casoni, M. Badea, N. Olah, C.S. Cobzac

**Affiliations:** 1 Babes-Bolyai University, Cluj-Napoca, Romania; 2 Transilvania University of Braşov, Braşov, Romania; 3 "Vasile Goldis" Western University of Arad, Faculty of Pharmacy, Arad, Romania, SC PlantExtrakt SRL, Rădaia, Cluj, Romania

The herbal remedies, including *Eugenia cariophillata* (cloves), have a popular usage as an alternative to the allopathic medicine. Hydroalcoholic extracts of clove were obtained using four different extraction systems: water (E1); ethanol-water (1:1, v/v) (E2); ethanol-water (7:3, v/v) (E3) and ethanol (E4). The extracts were obtained using classical (maceration-M and reflux-R) and modern techniques (ultrasonication-UAE and microwave assisted extraction performed in two different sets of parameters: 30 seconds at 700 W and 60% duty coefficient -MAE1 and 60 seconds at 900 W and 40% duty coefficient - MAE2). In all the cases, the extraction ratio was 0.5g plant powder/20 mL solvent. The extracts were characterised through their content of flavonoids and polyphenols and antioxidant capacity (FRAP and SNP). 

The richest extract in flavonoids was obtained by using UAE and E2 extraction system (5.53μg rutine/mL). High quantities of extracted polyphenols were obtained performing UAE (135.3μg cafeic acid/mL), maceration (130.7μg/mL) and MAE2 (122.4μg/mL). It was observed that FRAP activity vary in the same manner with polyphenols content. Regarding SNP antioxidant capacity, the best value (19.81 µg trolox/mL) was obtained when MAE2 and E2 were used for extraction.

In conclusion, the study showed that higher antioxidant capacity and extraction yields were obtained when M, UAE and MAE2 were used. By comparing the extraction systems, the extracts obtained using E3 contain large amounts of polyphenols, having a higher FRAP antioxidant capacity. By using the E2 extraction system, flavonoids extraction is favoured and higher SNP antioxidant capacities were obtained.

# Meeting report: P.2.25. ANTIOXIDANT CAPACITY OF SOME GLYCEROL EXTRACTS OF PLANTS FROM ROSACEAE FAMILY

**Authors:** D. Casoni, M. Badea, N. Olah, C.S. Cobzac

**Affiliations:** 1 Babes-Bolyai University, Cluj-Napoca, Romania; 2 Transilvania University of Braşov, Braşov, Romania; 3 "Vasile Goldis" Western University of Arad, Faculty of Pharmacy, Arad, Romania, SC PlantExtrakt SRL, Rădaia, Cluj, Romania

Glycerin extracts of *Crategus* (C), *Rosa canina* (RC), *Rubus fructicus* (RF), *Rubus ideaus* (RI) undergrowth and *Prunus amygdalus* (PA), *Prunus spinosa* (PS), *Sorbus domestica* (SD) buds were investigated to determine the content of bioactive compound and antioxidant capacity. The content of Polyphenols and total flavonoids was determined based on Folin-Ciocaltau method and AlCl3 respectively. Antioxidant capacity (AC) was determined both by using FRAP and SNP methods. 

RC extract presents the highest antioxidant capacity (SNP) and content of polyphenols and flavonoids. By plotting flavonoids content vs. polyphenols content, the extracts could be classified in three groups: (i) SD-RF-RC (R2=0.9909); (ii) RI-PS-C (R2=0.5265); (iii) PA. The same classification could be observed in the case of flavonoids content vs. AC(SNP): (i) SD-RF-RC (R2=0.9948); (ii) RI-PS-C (R2=0.9999); (iii) PA, as well as for flavonoids content vs. AC(FRAP): (i) SD-RF-RC (R2=0.9890); (ii) RI-PS-C (R2=0.9064); (iii) PA. Polyphenols vs. AC(FRAP) plot shows a different classification of samples: (i) SD-PA-C (R2=0.9999); (ii) PS-RI-RF (R2=0.9942); (iii) RC. A direct correlation between polyphenols content and AC(SNP) for all samples (R2=0.9366) was observed. 

Following this study, two conclusions could be drawn: (i) the antioxidant capacity determined by SNP method can be predicted by the polyphenols content and (ii) the samples could not be classified based on the part of the plant which was subjected to extraction.

# Meeting report: P.2.26. OXIDATIVE STRESS PARAMETERS IN RELATION TO TOTAL ANTIOXIDANT ACTIVITY OF VEGETABLE OILS - MICROWAVE HEATING

**Authors:** L. Iosif, L. Gaman, M. Gilca, M. Radoi, V. Atanasiu, M. Iancu, I. Stoian, D. Dragos

**Affiliations:** 1 Carol Davila University of Medicine and Pharmacy, Bucharest, Romania; 2 R&D Irist Labmed, Bucharest, Romania

Microwave heating is a common cooking procedure. Heating can accelerate oxidative processes in oil sand oxidation products are known to have potential toxic effects on human health. Therefore it would be useful to have a method to anticipate the vegetable oils behaviour under thermal conditions. Several methods to evaluate total oxidant activity were developed but unfortunately their ability to predict the oxidative changes during heating are poorly estimated. In this study we evaluated the relation of TEAC FRAP and CUPRAC values together with total polyphenols and flavonoids content of several commercial available vegetable oils with conjugated dienes and TBAR’s products during 15 minutes microwave heating. FRAP values were correlated with oxidative parameters after 15 minutes heating.

# Meeting report: 3. New Trends in Engineering Sciences applied in Life Sciences KN.3.1. PULSED LASER DEPOSITION OF BIOMATERIAL FILMS: A USEFUL TECHNIQUE FOR STRUCTURE-COMPOSITION-PROPERTY STUDIES

**Authors:** V. Craciun, D. Craciun, O. Fufa, P. Garoi, G. Dorcioman, L. Floroian

**Affiliations:** 1 National Institute for Lasers, Plasma and Radiation Physics, Magurele, Romania; 2 Dentix Millennium SRL, Sabareni, Giurgiu, Romania; 3 Transilvania University, Brasov, Romania

The pulsed laser deposition (PLD) is probably the most versatile laboratory technique to grow thin films in order to investigate the interdependence between structure, composition and properties. Simple changes of the deposition conditions (substrate temperature, nature and pressure of the gaseous atmosphere, laser fluence, repetition rate, laser wavelength and deposition geometry) will result in the growth from a single target of films having a rather wide range of compositions, crystalline grain sizes, textures, stress levels and properties. 

We investigated the use of the PLD technique to deposit new materials that could be used as protective coatings for implants used in medicine.

We used the PLD technique to deposit various transition metal carbides, nitrides and oxides thin films. The technique allows for deposition of high quality coatings at moderate temperatures that exhibit good mechanical and chemical properties, are adherent, dense and free of cracks. 

SiC, ZrC, ZrN, TiN, Ti, and TiO2 films having various structures, from amorphous to polycrystalline were obtained. SEM and AFM investigations showed that depending on the target material and deposition conditions films that are smooth or rough could be obtained. Nanoindentation results showed that very hard films, much harder than the Ti implants could be obtained by the PLD technique. Films were very adherent and also exhibited low friction coefficients, an advantage when the implants are mobile. Also the films exhibited lower dissolution rates in simulated body fluids than pure Ti.

The results clearly showed the advantages of the PLD technique to obtain new and improve materials for biocompatible protective coatings. Coatings possessing very different chemical and mechanical properties could be easily obtained and characterized. The properties could be easily tuned for specific applications, where either good mechanical properties or low dissolution rates are required.

PLD is an excellent technique to grow biocompatible coatings for medical implants. By selecting specific irradiation regimes, films that are either amorphous or polycrystalline could be obtained. The mechanical properties could be improved by controlling the grain size and the chemical composition. Films are also very dense, exhibiting low dissolution rates and protecting the implant from the corrosive action of body fluids.

*This work was supported by grants of the Romanian Space Agency, ROSA STAR project numbers 60 and 65, UEFISCDI project PCCA 226, Project MIFID ID-P_39_366 and NUCLEU INFLPR program*.

# Meeting report: KN.3.2. BIG-DATA ANALYTICS AND USE OF UNMANNED AIRCRAFT SYSTEMS (UAS) IN CROP AND NUTRITION MANAGEMENT

**Authors:** G. C. Bora

**Affiliations:** 1 Agricultural and Biological Engineering Department Mississippi State University, USA

Information and Data are key in precision agriculture (PA), which involves the technique of applying the right amount of inputs such as seeds, water, fertilizer and chemicals at the right location and at the right time to enhance production with positive environmental impact. Sensors and controllers, telematics, and unmanned aerial system (UAS) are major source of spatial and temporal data in crop management to maintain proper nutritional balance. With major advances in field and equipment-based sensor technology and very high resolution remote sensing data by UAS; the potential to revolutionize food production rests on successfully addressing a number of challenges relating to acquiring, understanding, and using Big Data (BD). There are plenty of management issues with large UAS aerial imagery in terms of volume, data transfer, telematics, software availability, storage, security of storing in clouds, end uses besides the legal issues. There is also a need to increase the skilled workforce in this area. This study tried to address to find a solution for big-data management and apply information to optimize inputs for better nutritional value in food grain.

# Meeting report: O.3.1. ALDEHYDE DEHYDROGENASE FROM A PHYSCHROPHILIC BACTERIUM FOR COLD SENSOR APPLICATIONS

**Authors:** G. Necula-Petrareanu, P. Lavin, A. Vasilescu, M. Lapauw, C. Purcarea

**Affiliations:** 1 Institute of Biology of the Romanian Academy, Bucharest, Romania; 2 Instituto Antofagasta, Universidad de AntofagastaChile; 3 International Centre of Biodynamics, Bucharest, Romania,; 4 Universite de Perpignan Via Domitia , Perpignan,France

Extremozymes, enzymes found in microorganisms able to tolerate extreme conditions, are one of the recently used solutions for obtaining improved biosensors used in industrial processes. A challenge for measuring the content of various aldehydes in the wine industry represents the development of stable and highly sensitive aldehyde dehydrogenase (ALDH)-based biosensors. Therefore, we are interested in developing cold-active biosensors using a recombinant ALDH from *Flavobacterium* PL02, a phychrophilic bacterium isolated from Antarctic sea water. Structural analyses of the putative enzyme revealed the conservation of active site residues and an amino acid composition common to cold adapted proteins, suggesting an active catalyst stable at low temperatures. Three dimensional structure modelling indicated a homologous folding with Staphylococcus aureus ALDH. The gene coding for this ALDH was cloned in the pHAT2 expression vector, and expressed in E. coli as soluble protein (F-ALDH) by IPTG induction for 16 hours at 25°C. The recombinant F-ALDH was purified in one step by Ni-affinity chromatography. Activity measurements at 30°C indicated the utilization of benzaldehyde as substrate and NAD+ as cofactor. Enzymatic analyses of F-ALDH are currently under way, in order to evaluate the functional properties of this enzyme, as a prerequisite for developing biosensors for aldehyde content monitoring.

*This work was financially supported by UEFISCDI, project PN-III-P2-2.1-PED-2016-0116 (contract 1PED/2017)*. 

# Meeting report: O.3.2. ENZYMATIC BIOFET TRANSISTOR PROCESSING IN A MICROELECTRONIC FOUNDRY

**Authors:** C. Ravariu, F. Babarada, C. Pirvulescu, E. Manea

**Affiliations:** 1 University Politehnica of Bucharest, ETTI, DCAE, Bucharest, Romania,; 2 National Institute of Microtechnology, Bucharest, Romania

The Bio-FET transistors are complex electronic devices that integrate in the same chip a Field Effect Transistor (FET) with a sensitive biological receptor. This work starts to present the TCAD techniques used for the simulation and design of the BioFET device, followed by a specialised software ATLAS from Silvaco useful for the physical phenomen an investigation and finishing by few main technological steps that are applied for enzymatic BioFET transistor processing in a microelectronic foundry. By some EDA tools as LEDIT, the layers that will constitute the fabrication masks will be established; then they are transposed on a glass support with chromium metallic traces. The masks and values of the designed parameters have to be correlated with the process fabrication and device size. By some EDA tools as LEDIT, the layers that constitute the fabrication masks that are established; then they are transposed on a glass support with chromium metallic traces. The biodevice description in Atlas program, include an original approach concerning the simulation techniques of the enzymes entrapping onto nanostructured material grown on Silicon wafer.

An enzymatic transistor was implemented in Si, appealing to multiple facilities from a Microelectronics foundry. 

# Meeting report: O.3.2. DEVELOPMENT OF A VERSATILE SPR APTANSENSOR FOR DETECTION OF TRACES OF LYSOZYME DIMER IN OLIGOMERIC AND AGGREGATED MIXTURES

**Authors:** A Vasilescu, C. Purcarea, E. Popa, M. Zamfir, I. Mihai, S. Litescu, S. David, S. Gaspar, M. Gheorghiu, JL Marty

**Affiliations:** 1 International Centre of Biodynamics, Bucharest, Romania; 2 Institute of Biology of the Romanian Academy, Bucharest, Romania;; 3 University of Bucharest, Faculty of Chemistry, Bucharest, Romania;; 4 National Institute for Research and Development in Biological Sciences, Bioanalysis Center, Bucharest, Romania;; 5 BAE: Biocapteurs-Analyses-Environnement, Universite de Perpignan Via Domitia, Perpignan, France

Protein aggregates formed in various steps during the manufacturing or storage of therapeutic proteins affect the quality, safety and efficacy of biopharmaceutics [1]. Working towards developing a sensitive method that will allow detecting low amounts of oligomers in concentrated protein solutions, as an alternative to the current separation-based procedures, we have optimised an aptasensor with SPR detection [2] for the detection of lysozyme dimer- chosen here as model of protein oligomers. The method exploits the differences in binding kinetics and affinity between the lysozyme monomer and dimer, to an aptamer developed for the monomeric form. By analysing the sensorgrams via chemometrics, the SPR aptasensor allowed determining 0.1-1% dimer in solutions of lysozyme monomer. Moreover it was applied to observe the variations in lysozyme oligomer amounts during the aggregation of lysozyme solutions at 60°C and pH 2. The study could serve as a model for the sensitive detection of small oligomers in therapeutic proteins, for which specific aptamer exists, thus circumventing the need for chromatographic separation. The versatile aptasensor can be tuned, by simply adjusting the experimental conditions, for the sensitive and specific detection of either the monomer or the dimer, as per the desired application.

# Meeting report: O.3.3. EVALUATION OF CARBON-NANOMATERIAL BASED SENSORS FOR NADH DETECTION

**Authors:** A. Vasilescu, M. Lapauw, G. Necula-Petrareanu, C. Purcarea

**Affiliations:** 1 International Centre of Biodynamics, Bucharest, Romania; 2 Université de Perpignan Via Domitia, Perpignan, France;; 3 Institute of Biology of the Romanian Academy, Bucharest, Romania

Electrochemical detection of NADH based on its direct oxidation on screen-printed electrodes registered significant advances with the emergence of nanomaterials [1,2] with regards to sensitivity and decrease in oxidation potential. Highly performant yet low cost NADH sensors continue to be actively sought for their potential applications in biosensors with more than 250 NAD+-dependent dehydrogenases. Combining nanomaterials with electrochemical mediators on screen-printed electrodes allows sensitive detection of NADH at low potentials in the range of minimal interferences typically encountered in real-world samples [3]. Hereby we investigated the utility of a known electrochemical mediator for NADH, Meldola Blue with various commercial screen-printed carbon electrodes, i.e. with mesoporous carbon, modified with single or multi wall carbon nanotubes or modified with carbon nanofibers. These electrodes were compared with commercial Meldola Blue-modified electrodes. Meldola Blue/carbon nanofibers electrodes allowed to achieve a detection limit of 1.0 µM NADH and a sensitivity of 13.8 ±1.1 µA.Lmmol-1 by amperometry in 0.1 M PBS buffer pH 7.4 and at 25 mV. The utility of Meldola-Blue modified electrodes as NADH detectors with NAD+-dependent aldehyde dehydrogenase was also explored, in view of further applications in single-use biosensors for dithiocarbamate fungicides, based on the principle of enzymatic inhibition. 

*A.V, G.N-P and C.P acknowledge the financial support by the Executive Agency for Higher Education, Research, Development and Innovation Funding (UEFISCDI), Romanian Ministry of Scientific Research and Innovation, project PN-III-P2-2.1-PED-2016-0116*.

# Meeting report: O.3.4. IMPROVEMENT OF THE ADHESION BETWEEN HYDROXYAPATITE AND TI6AL4V ALLOY USED FOR BIOMEDICAL APPLICATIONS

**Authors:** M. Dinu, V. Pruna, M. Braic, C.M. Cotrut, I. Titorencu, I. Pana, V. Braic, A. Vladescu

**Affiliations:** 1 National Institute for Optoelectronics, Magurele, Romania; 2 Institute of Cellular Biology and Pathology "Nicolae Simionescu" of the Romanian Academy, Bucharest, Romania;; 3 University Politehnica of Bucharest, Bucharest, Romania

Poor adhesion between the orthopaedic implants and the surrounding tissue was already validated in biomedical applications. To tackle this issue, bioactive coatings based on calcium phosphates are already used in reconstructive surgery, due to their chemical similarity with bone matrix. 

The work presented tackles the hydroxyapatite (HAP) adhesion to T6Al4V alloy, by using a carbide coating as adhesion interlayer for the top HAP coating. Several transition metal carbide (TiC, ZrC and NbC) were selected in this study, due to their enhanced adhesion to metallic substrates, anticorrosive properties and biocompatibility.

The coatings were prepared by magnetron sputtering technique and were investigated in terms of elemental and phase composition, topography, roughness, hardness and elastic modulus, and particularly adhesion. Since these films are expected to coat metallic orthopaedic implants, their resistance to the SBF corrosive attack and the cytotoxicity were also evaluated.

The results revealed an improved adhesion between Ti6Al4V alloy and HAP coatings by the use of these carbide interlayers, with no negative influence on the biomedical properties, such as good corrosion resistance, low elastic modulus, high specific cells proliferation and gene expression levels. Moreover, the observed formation of the HAP crystalline structure indicated that whatever the carbide adhesion layer, no modification of HAP composition and stoichiometry were evidenced. The comparative investigation of the three types of interlayer demonstrated that NbC interlayer represents the best solution for improving the adhesion between the HAP coating and Ti6Al4V alloy substrate, suggesting a possible enhancement in service life of such coated orthopaedic implants.

The work was supported under the grant of the Romanian National Authority for Scientific Research, CNCS – UEFISCDI, project number PN-II-PT-PCCA-2014-212 (OSSEOPROMOTE). SEM, EDS & XRD analyses were carried out using the equipment acquired by the infrastructure project INOVA-OPTIMA SMIS code 49164, contract no. 658/2014. 

# Meeting report: P.3.1. DOUBLE NANOSTRUCTURED LAYERS FOR BIOMIMETIC IMPLANT APPLICATIONS

**Authors:** L. Floroian, G. Puchianu, A. Manea, M. Gilca, M. Badea

**Affiliations:** 1 Transilvania University of Brasov, Brasov, Romania;; 2 "Carol Davila" University of Medicine and Pharmacy, Bucharest, Romania

Ayurvedic medicine is one of the world’s oldest medical systems. It is an example of a coherent traditional system which have a time tested and precise algorithm for medicinal plant selection, based on several ethno-pharmacophore descriptors whose knowledge endows the user to adequately choose the optimal medicinal plant for the treatment of certain pathology.

This work tries to link traditional knowledge with biomedical science by using traditional ayurvedic plants with antimicrobial effect in manufacture of thin films for implant protection.

We report on the transfer of novel polymer- ayurvedic extract-bioactive glass composites by matrix assisted pulsed laser evaporation to uniform thin layers onto dental implant. The targets were prepared by freezing in liquid nitrogen of mixtures containing polymer and ayurvedic extract, reinforced with bioglass powders and then were submitted to multipulse ablation with an UV KrF* (λ=248 nm, τ ~ 25 ns) excimer laser source. The behaviour of polymer - ayurvedic extract – glass / implant structure in condition which simulates the physiological environment was evaluated in vitro by complementary techniques.

When in contact with body fluids, the coatings demonstrate the ability to stimulate the growth of biological hydroxyapatite on their surface, which validates the film bioactivity. Bioglass dissolution in human body fluids is accompanied by a prolonged release of antibiotic active molecules, an ideal circumstance for prevention of local infections. 

Both polymer and apatite layer that grows on the implant surface four weeks after samples introduction into simulated body fluid, ensure a good protection against degradation and release of harmful metallic ions into the body. The printed structures are highly biocompatible and resistant to microbial colonization and induce a significant delay in the microbial biofilm initiation and their further development.

The results emphasize the multiple advantages of these coatings which would allow to halt any leakage of metal and metal oxides to the biological fluids and eventually to inner organs (by polymer use), to speed up the osseointegration (due to the bioactive glass use) and to exert antimicrobial effects (by ayurvedic plants extracts use).

*This work was supported by a grant of the Romanian National Authority for Scientific Research and Innovation, CNCS/CCCDI UEFISCDI, project number PN-III-P2-2.1-PED-2016-0621, within PNCDI III*.

# Meeting report: P.3.2. NUCLEAR THERAPY: OUR MAIN WEAPON AGAINST CANCER

**Authors:** D. Calaver, L. Floroian

**Affiliations:** * Transilvania University of Brasov, Brasov, Romania

Nuclear therapy uses different methods to physically kill certain cells using some nuclear radiation, radiotherapy use electro-magnetic radiation instead and other methods are bio-chemically-based. 

This is a systematic review of numerous studies regarding cancer treatments and we are looking at possible synergies between these methods to obtain an optimal effect. 

Boron Neutron Capture Therapy (BNCT), radiotherapy, chemotherapy and surgery are methods already tested with patients and gene-engineering with Gadolinium neutron capture therapy (Gd-NCT) are methods that still need more data. The median survival times of patients in the BNCT are similar to that of the conventional beam X-irradiation, when used together the median survival time doubled in some cases. For the other methods more studies must be made before using clinically.

BNCT or Gd-NCT can be used to target cancer cells and so has few effects on healthy tissues; radiotherapy may have a stronger effect but can’t make the difference between healthy and cancer cells; chemotherapy try to affect the way patients’ cells divide and because the division rate of cancer cells is greater than of the healthy cells they will be more affected (but again, this also affect healthy tissues); by surgery we can remove a big part of a tumor, and, gene-engineering could improve our immune system.

If we add these together the patients may have a greater chance of survival. The main problems in these therapies remain the separation of healthy cells from the cancer cells.

# Meeting report: P.3.3. ELECTRO ASSISTED BSA INTERFACE: TOWARDS A PERFECT ANTIFOULING SURFACE FOR BIOMEDICAL SENSING APPLICATIONS

**Authors:** M. H. Nawaz, G. Catanante, J- L. Marty, A. Hayat

**Affiliations:** 1 Interdisciplinary Research Centre in Biomedical Materials, COMSATS Institute of Information Technology, Lahore, Pakistan; 2 BAE Laboratory, Université de Perpignan Via Domitia, Perpignan, France

The aim of the present study was to investigate the direct electrooxidation of BSA without stabilizer and adsorbent, onto the carbon electrode at various protein concentrations and pH buffer solutions. A simple strategy based on electro oxidation of BSA on to the working electrode of carbon based screen printed electrode, gave a good immobilization support for biological molecules. The study anticipated that this surface not only provide antifouling characteristics, but it can also result into a good conducting transducer interface. Moreover, due to presence of other reactive groups, BSA can provide a soft platform for further surface functionalization. It may also facilitate to understand the proteins interactions with artificial surfaces, selective oxidation within the macromolecular domains and analytical separation.

# Meeting report: P.3.4. A FUZZY-LOGIC BASED MODEL OF INSULIN-TO-GLUCOSE SYSTEM WITH USE IN GLYCAEMIA AUTOMATIC CONTROL SIMULATION STUDIES

**Authors:** C. Boldișor, S. Coman, L. Floroian

**Affiliations:** 1 Automatic Control and Information Technology Dept., Transilvania University of Brașov, România

Several models describing the insulin-to-glucose dynamics in type-1 diabetes mellitus have been intensively used in simulation studies, in search of reliable and efficient control algorithms to be used in automatic treatment. Some models are appropriate for designing control algorithms, but lack in catching process’ real dynamics. Other models allow more precise simulations, but their complexity makes them difficult to use in control engineering. 

We review a fuzzy logic based model of the insulin-to-glucose system derived from the intensively used non-linear minimal model. Our model allows designing control algorithms using methods from control engineering and it simulates the glycaemia dynamics more precisely than the usual linearized minimal model. 

The model is derived through a variable substitution in the original model, which is correct in the sense of the fuzzy logic. We implemented it in a Matlab program, with which we compare the glycaemia dynamics for a few variants of the basic model. 

The simulation results (glycaemia time-responses under exogenous inputs of insulin or glucose) consist of charts and summarized tables. The results show that the time-response (glycaemia dynamics) of the model we derived is much closer to the original one than the linear models used in many simulation studies.

Presented fuzzy model imitates the glycamia dynamics better than the linearized models used in many articles, both under exogenous input of insulin or under glucose disturbance input. The model can be easily used in designing and simulating fuzzy logic control algorithms for glycaemia automatic control.

# Meeting report: P.3.5. HYDROXYAPATITE COATINGS WITH SiC AND Mg ADDITION FOR BIOMEDICAL APPLICATIONS

**Authors:** M. Dinu, R. Abdulgader, M. Braic, R. Booysen, V. Braic, T.K. Monsees, A. Vladescu

**Affiliations:** 1 National Institute for Optoelectronics, Magurele, Romania; 2 Department of Medical Biosciences, University of the Western Cape, Bellville, South Africa

A good bonding between the metallic implants and bone is needed for a long-lasting implant. Up to now, cemented prostheses were used. However, the cement can breakdown, leading to a weakening of joint and inflammation due to as-generated debris. To avoid rejection of the artificial joint implant or possible revision surgery, the present research is focused on improving the strength of the metallic implant-tissue interface. By coating of the implant surface with bioactive coatings, the osseointegration of the implants can be promoted. Moreover, an addition of SiC and Mg is believed to decrease the dissolution rate of hydroxyapatite (HAP) coatings, without losing its osseoconductive ability.

The HAP coatings with additions of SiC and different Mg amount were deposited on Ti6Al4V substrates by RF magnetron sputtering technique. Thus, it is expected that the Mg addition into the Si doped HAP will combine the mechanical properties of HAP+Si coatings with the good osseointegration capability of Mg. The evaluation of the coated specimens was performed in terms of elemental composition and chemical bonding, morphology and electrochemical behaviour. Additionally, a special attention was given to mechanical and biological investigations. 

The results showed that the mechanical properties, corrosion resistance and degradation rate of HAP-SiC was significantly improved by Mg addition. 

To authenticate the biocompatibility of prepared coatings, SaOS-2 bone cells attachment and growth studies were carried out, indicating that the novel coatings are compatible with biomedical applications, while improving also the mechanical properties of HAP-SiC reference coating. 

*The work was supported by a grant of the Romanian National Authority for Scientific Research and Innovation (CNCS/CCCDI–UEFISCDI), project number PN-III-P2-2.1-PED-2016-1854, within PNCDI III (117PED-DegraCoat)*.

# Meeting report: P.3.6. USING SIMPLELINK WIFI BOARD TO IMPLEMENT INTERNET OF THINGS

**Authors:** D. Floroian

**Affiliations:** * Transilvania University of Brașov, Brașov, România

IoT based smart home was accomplished through a hardware – software device which is founded on revolutionary technology Internet of Things (IoT) and focuses on portability and flexibility.

The created device distinguishes from other existing equipment on the market due to the fact that it connects through wireless and has a very low power consumption.

The whole system consist of the user’s smartphone or tablet, the house existing router, the development board SimpleLink Wi-Fi CC3200, sensors and a relay board. 

In order to realise communications the actual internet infrastructure is being used, thus from the house the connection to the created device is being established through the router and from anywhere else the connection to the device is realised through cloud which communicates with the router and the last one communicates with the development board.

# Meeting report: P.3.7. HARDWARE INTERFACE FOR INTERNET OF THINGS APPLICATIONS

**Authors:** D. Floroian

**Affiliations:** * Transilvania University of Brașov, Brașov, România

We are living in „quickness century” and it happens uncountable times when leaving the home to forget the lights turn on. By using the smartphone, the client can switch off the lights from every corner of the world. 

The work presents the connections that have to be done in order to connect the relay board at the Launchpad, thus the relay board takes GND and VCC from CC3200 and Relay1, Relay2, Relay3 and Relay4 are the GPIO pins that provides 0 logic value in order to activate the correspondent relay or 1 logic value to deactivate it.

By using the actual internet infrastructure which allows things to exchange data without requiring human intervention, by using hardware which made possible the capability to retrieve data from things and software which enable data instruction and manipulation, the requirements were successfully accomplished.

# Meeting report: P.3.8. CLOUD HEALTH MONITORING SYSTEM

**Authors:** C. I. Goran, C. Gerigan

**Affiliations:** * Transilvania University of Brașov, Brașov, România

A health monitoring system based on a poor social environment where not everyone can afford expensive products which can monitor your vital signs or smartphones with advanced health monitoring systems.

We can use cheap smartphones and digital wristbands that can gather all data we need to anticipate health anomalies using a smart algorithm.

The Cloud Health Monitoring System is based on a simple fitness wristband, an IOS or Android application and internet connectivity. The cloud computing service is covering data processing and administration. The other devices are only used for data gathering for battery saving purposes. 

Two devices cheap and easy to find, connected to a cloud server helped by a 24/24 continuous process witch constantly verifies data from the hearth rate monitor sensor, gyroscope sensor and GPS.

Every cloud account can be managed by the family doctor. He can download statistics, push manual health warning notifications and live update for the health government insurance card. 

We use devices that can provide us valuable data. The synergy between them can anticipate health problems earlier with a minimum price for reliable software.

# Meeting report: P.3.9. BIOCHEMICAL CHANGES INDUCED IN VIVO BY SI/SIO IN LIVER TISSUE

**Authors:** D.A. Nita, M. Stan, A. Dinischiotu

**Affiliations:** * Department of Biochemistry and Molecular Biology, University of Bucharest, Romania

Quantum dots (QDs) are nanocrystalline semiconductor materials that have been recently tested for biological applications such as cancer therapy, cellular imaging and drug delivery. The purpose of this study was to evaluate in vivo the degree of oxidative stress generated at the liver level following administration of Si / SiO2 QDs.

Silicon QDs toxicity was investigated by injection into the codified vein of these Si / SiO2 QDs in Swiss mice, being tested in 3 different concentrations (1, 10 and 100 mg QDs / kg body weight). After 24 hours of nanoparticle administration, the mice were sacrificed and liver tissue was sampling. From the total protein extracts, were measured the specific activities of the antioxidant enzymes (superoxide dismutase (SOD), catalase (CAT), glutathione peroxidase (GPX), glutathione reductase (Gred), glutathione S-transferase (GST), glucose 6-phosphate dehydrogenase (G6PDH), as well as reduced glutathione (GSH) and malonaldehyde (MDA) concentration, the results have been reported to mice injected with phisiological serum. 

The analyzes showed that the highest dose (100 mg QDs / kg body weight), 30% decrease in CAT activity, 22% G6PDH activity, 15% GST activity, and 20% GPX and GSH concentration. The determinations performed demonstrate the lack of toxicity of Si / SiO2 QDs to concentrations of 10 mg/kg body, not affecting the redox balance at the liver.
